# Exogenous ABA enhances cold tolerance of *Rhododendron yedoense* var. *poukhanense* under subzero temperature: integrating physiology, transcriptome, and proteome

**DOI:** 10.3389/fpls.2026.1921248

**Published:** 2026-08-28

**Authors:** Riwen Fei, Jiayi Liu, Siyang Duan, Minghang Guo, Hongmei Zhu, Yanhong Zhang, Xiuting Zhao, Jiansong You, Xiaoyu Li

**Affiliations:** 1 School of Agricultural, Liaodong University, Dandong, China; 2 Postdoc Workstation, Aim Honesty Biopharmaceutical Co., Ltd., Dalian, China; 3 School of Bioengineering, Dalian University of Technology, Dalian, China

**Keywords:** abscisic acid, cold stress, physiological property, proteomics, Rhododendron yedoense var. Poukhanense, transcriptomics

## Abstract

Low temperature limits the growth and ornamental value of evergreen shrubs. *Rhododendron yedoense* var. *poukhanense*, an important ornamental shrub from Northeast China, frequently suffers freezing damage during winter. While exogenous abscisic acid (ABA) enhances cold tolerance in many plants, its molecular mechanisms at subzero temperatures remain poorly understood in non-model species lacking chromosome-level reference genomes. This study investigated the effects of exogenous ABA on freezing tolerance in *R. yedoense* var. *poukhanense* at -4 °C using an integrated physiological, transcriptomic, and proteomic approach. Cutting seedlings were subjected to four treatments: CK (22°C control), A (22°C + ABA), LT (-4°C), and ALT (-4°C + ABA). Photosynthetic pigments, osmotic regulation substances, antioxidant enzyme activities, and malondialdehyde (MDA) content were measured. Transcriptome sequencing and quantitative proteomics were performed, and transcriptome data were validated by quantitative real-time PCR (qRT-PCR) of 15 selected genes. ABA pretreatment reduced visible cold injury severity, partially preserved photosynthetic pigments, decreased MDA content by 28.7%, and promoted recovery of catalase (+43.6%), superoxide dismutase (+31.1%), and peroxidase (+20.0%) activities under freezing stress. Transcriptome analysis revealed 8, 444 differentially expressed genes (DEGs) in LT versus CK and 6, 481 DEGs in ALT versus CK, representing a 23% reduction in transcriptional reprogramming scope attributable to ABA priming. The ALT versus LT comparison identified only 1, 690 additional DEGs, indicating that most cold-responsive genes were pre-activated during the ABA priming phase. Proteome analysis identified 1, 461 differentially expressed proteins (DEPs) in ALT versus CK. Integrated analysis revealed extensive post-transcriptional regulation, with transcript-protein concordance of only 1.0-4.1%, and co-enriched Kyoto Encyclopedia of Genes and Genomes (KEGG) pathways in both omics layers. qRT-PCR validation confirmed high reliability of the transcriptome data (R^2^ = 0.8500). These findings demonstrate that exogenous ABA enhances freezing tolerance through multi-layered molecular regulation encompassing transcriptional buffering, translational reprogramming, and functional reallocation from photosynthesis to stress protection. This study provides the first integrated physiology-transcriptome-proteome framework for ABA-mediated freezing tolerance in an evergreen ornamental shrub and offers theoretical support for ABA-based winter protection strategies.

## 1 Introduction

Low temperature is one of the most common abiotic stresses that limit plant growth, spread, and farm output worldwide (Thomashow, 1999). When plants face low temperatures, they go through harmful body changes. These include blocked photosynthetic electron transport, damaged membrane lipid fluidity, buildup of reactive oxygen species (ROS), and upset cell metabolic balance (Thomashow, 1999). These effects cause further damage, such as protein breakdown, electrolyte leakage, and broken energy metabolism. For evergreen woody shrubs that keep their leaves all winter, the ability to survive freezing temperatures is very important, as leaf tissues must maintain functionality during cold acclimation processes that involve structural and physiological adaptations (Wang et al., 2008; Marian et al., 2004).

The genus *Rhododendron* (Ericaceae) has over 1, 000 species. They are found across temperate and subarctic East Asia. Many are used as model systems for cold hardiness research (Arora and Rowland, 2011). In *R. catawbiense*, the dehydrin protein RcDhn5 was shown to improve freezing tolerance when overexpressed in transgenic *Arabidopsis*. And this set a direct link between dehydrin buildup and cryoprotection (Peng et al., 2008). Antioxidant enzyme networks (superoxide dismutase, SOD; catalase, CAT; peroxidase, POD) as key parts of cold defense (Mittler, 2002). Transcriptome studies of *R. aureum* showed large changes in photosynthesis, hormone signal transduction, and membrane lipid remodeling genes (Cao et al., 2022). And integrated multi-omics in *R. chrysanthum* showed that ABA synthesis, MAPK cascades, and Ca^2+^ signaling work together to control cold tolerance. They do this by adjusting stomatal closure, chlorophyll breakdown, and ROS balance (Chen et al., 2024). But even with these findings, ABA-mediated freezing tolerance mechanisms are still not fully understood in *Rhododendron*. And this is especially true under subzero conditions.

*Rhododendron yedoense* var. *poukhanense* is an important evergreen ornamental shrub from northeastern China and the Korean Peninsula. It is widely grown in Heilongjiang, Jilin, and Liaoning provinces. People like it for its glossy dark-green leaves and lots of spring flowers. Field studies show this plant can survive temperatures down to about -30°C when it is fully dormant. But its evergreen leaves still get hurt by freezing during mid-winter warm spells followed by sudden cold. This plant has great garden value. But the body and gene mechanisms that control its freezing tolerance have not been fully studied. And no one has tested whether outside hormone treatments can help it resist cold better.

Abscisic acid (ABA) is a sesquiterpenoid phytohormone that controls how plants respond to many abiotic stresses. These include drought, salinity, and low temperature (Chinnusamy et al., 2007). When plants sense cold, ABA levels go up briefly. And this happens through higher expression of *NCED*, which is the rate-limiting enzyme in ABA biosynthesis. Built-up ABA turns on a standard signaling path. This path has PYL receptors, group A PP2C phosphatases, and SnRK2 kinases (Chinnusamy et al., 2007). When ABA is present, PYL receptors bind and block PP2C phosphatases. And this frees SnRK2 from repression. Then activated SnRK2 adds phosphate groups to ABF transcription factors. And these move to the nucleus. There they turn on stress-responsive gene expression by binding ABREs in target gene promoters (Chinnusamy et al., 2007). Cold-induced leaf senescence is accompanied by membrane leakage and altered antioxidant enzyme activities (Dhindsa et al., 1981), while the ABA-mediated ICE1-CBF-COR pathway. And this is the core transcriptional control axis for freezing tolerance. SnRK2.6/OST1 directly adds phosphate groups to ICE1 to make it more stable. And this helps ICE1 turn on *CBF* genes. ABF transcription factors also bind ABRE *cis*-elements in CBF and COR promoters. The SnRK family protein kinases, such as OsSAPK4 in rice, play crucial roles in stress signal transduction (Diedhiou et al., 2008), and exogenous ABA has been shown to improve cold tolerance in many plant species. In *Dendrobium officinale*, which was shown by higher proline and soluble sugar levels, lower MDA and H_2_O_2_ levels, and higher *PYL*, *PP2C*, *SnRK2*, and *ABF* expression (Wang et al., 2026).

New progress in fast sequencing and proteomics based on mass spectrometry has allowed people to study plant stress responses at the system level by combining multiple omics data. Gene profiling shows global gene expression changes. And quantitative protein analysis measures actual protein amounts. And this catches control steps after gene transcription. These steps often make protein levels different from gene levels (Pagano et al., 2021). In *Camellia japonica*, combined gene and protein analyses showed joint increases in starch and sucrose metabolism, glycerophospholipid reshaping, and antioxidant defense under low temperature (Guy et al., 2008). In spring wheat, combined multi-omics profiling showed that starch and sucrose metabolism play key roles in keeping pollen alive under cold stress (Zhao et al., 2019). Nevertheless, no investigation has applied an integrated physiology-transcriptome-proteome framework to elucidate ABA-mediated freezing tolerance mechanisms in any *Rhododendron* species, representing a significant gap in our understanding.

The present study was designed to address this knowledge gap by investigating the effects of exogenous ABA on cold tolerance in *R. yedoense* var. *poukhanense* at -4°C using an integrated physiological, transcriptomic, and proteomic approach. This study pursues four interrelated objectives. First, to characterize physiological responses, including photosynthetic pigment content, osmolyte accumulation, antioxidant enzyme activities, and membrane stability indicators, under low temperature and exogenous ABA treatment; second, to identify transcriptomic reprogramming events and DEGs via MGI-platform RNA-seq; third, to profile proteomic changes and DEPs via ultra-fast quantitative proteomics; and to construct an integrated ABA-mediated cold tolerance network through multi-omics correlation analysis. The four-treatment experimental design-comprising control (CK, 22°C), exogenous ABA alone (A, 22°C + 30 mg/L ABA), low temperature alone (LT, −4°C), and combined ABA plus low temperature (ALT, −4°C + 30 mg/L ABA)-enables systematic disentanglement of temperature effects, ABA effects, and their interactions.

## 2 Materials and methods

### 2.1 Plant materials and growth conditions

Healthy adult plants of *R. yedoense* var. *poukhanense* were taken from the Horticultural Facilities and Environment Practical Training Base of Liaodong College, Dandong, Liaoning Province, China (40°07′ N, 124°23′ E). Uniform one-year-old cutting seedlings were moved into plastic pots (diameter 15 cm, height 12 cm) with a sterilized mix of peat, perlite, and vermiculite (3:1:1, v/v/v). Plants were kept in a greenhouse for four weeks under these conditions: day temperature 20-25°C, night temperature 15-18°C, humidity 60%-70%.

### 2.2 Optimization of exogenous ABA concentration

A preliminary experiment was conducted to determine the optimal exogenous ABA concentration for improving cold tolerance. Four ABA levels were tested: 0 (deionized water control), 10, 30, and 50 mg·L^-1^. ABA (Sigma-Aldrich, St. Louis, MO, USA, Cat. No. A1049) was mixed in deionized water with 0.01% (v/v) Tween-20 as a surfactant. Cutting seedlings were sprayed on both leaf surfaces until water started to drip (~20 mL per plant) daily at 09:00 for 3 consecutive days in an artificial climate chamber (Ningbo Jiangnan Instrument Factory, PRX-450B, Ningbo, China) set at 22°C. The chamber was maintained with a 16 h light (PPFD 200 μmol·m^-2^·s^-1^)/8 h dark and 60%-70% relative humidity. On day 4, cutting seedlings were transferred to -4°C in the artificial climate chamber. Cold injury symptoms were monitored daily for 5 days. Based on phenotypic severity, 30 mg·L^-1^ was selected as the optimal concentration for the main experiment (**Supplementary Figure 1**).

### 2.3 Experimental design and treatments

A completely randomized design was established with four treatment groups. All groups underwent a 3-day pretreatment phase at 22°C followed by a 5-day treatment phase, with sampling at 0 d (day 4, before treatment) and 5 d (day 8, after treatment): (1) CK (Control) 22°C, deionized water spray; (2) A (ABA alone) 22°C, 30 mg/L ABA foliar spray; (3) LT (Low Temperature) -4°C, deionized water spray; (4) ALT (ABA + Low Temperature) -4°C, 30 mg/L ABA foliar spray. Each treatment comprised three biological replicates with five plants per replicate (n = 15). Fully expanded mature leaves were collected at 0 d (before treatment) and 5 d (after treatment), rapidly frozen in liquid nitrogen, and stored at -80°C until analysis.

### 2.4 Physiological index measurements

#### 2.4.1 Leaf pigment content

Chlorophyll a (Chl a), chlorophyll b (Chl b), and carotenoid contents were determined by UV spectrophotometry. Fresh leaf tissue (0.2 g) was ground in 10 mL of 80% (v/v) acetone with quartz sand and extracted in the dark at 4°C for 24 h. After centrifugation at 10, 000 × g for 10 min at 4°C, absorbance was measured at 470 nm, 646 nm, and 663 nm using a UV-visible spectrophotometer (Shimadzu UV-2600, Kyoto, Japan). Pigment concentrations were calculated using the equations of Lichtenthaler and expressed as mg/g fresh weight (FW).

#### 2.4.2 Soluble sugar, soluble protein, and proline content

Soluble sugar (SS) content was measured using a commercial kit (Solarbio Science & Technology Co., Ltd., Beijing, China, Cat. No. BC0030) by the anthrone-sulfuric acid colorimetric method. Fresh leaf tissue (0.1 g) was extracted in distilled water at 100°C for 10 min, centrifuged at 8, 000 × g for 10 min, and the supernatant was reacted with anthrone reagent at 95°C for 10 min. Absorbance was read at 620 nm.

Soluble protein (SP) content was determined by the Coomassie brilliant blue G-250 method. Fresh leaf tissue (0.2 g) was homogenized in 5 mL of 50 mM sodium phosphate buffer (pH 7.8) containing 1 mM EDTA and 1% (w/v) polyvinylpyrrolidone (PVP), then centrifuged at 12, 000 × g for 15 min at 4°C. Protein content was quantified using Coomassie brilliant blue G-250 (Solarbio, Cat. No. PC0010) with bovine serum albumin as the standard; absorbance was read at 595 nm.

Proline (Pro) content was assayed using a commercial kit (Solarbio, Cat. No. BC0290) following the acid-ninhydrin method. Fresh leaf tissue (0.2 g) was homogenized in 5 mL of 3% (w/v) sulfosalicylic acid and boiled for 10 min. After centrifugation at 10, 000 × g for 10 min, the supernatant was reacted with glacial acetic acid and acid ninhydrin at 100°C for 30 min. The product was extracted with toluene and absorbance measured at 520 nm.

#### 2.4.3 Antioxidant enzyme activities

Superoxide dismutase (SOD) activity was assayed using a commercial kit (Solarbio, Cat. No. BC0170) based on the nitroblue tetrazolium (NBT) photoreduction inhibition method. Fresh leaf tissue (0.2 g) was homogenized in ice-cold 50 mM sodium phosphate buffer (pH 7.8) and centrifuged at 12, 000 × g for 15 min at 4°C. One SOD unit was defined as the enzyme amount inhibiting 50% of NBT photoreduction.

Peroxidase (POD) activity was measured using a commercial kit (Solarbio, Cat. No. BC0090) by the guaiacol oxidation method. The reaction mixture contained enzyme extract, 50 mM sodium phosphate buffer (pH 7.0), 0.3% (v/v) H_2_O_2_, and 0.2% (v/v) guaiacol. Absorbance increase at 470 nm was recorded for 3 min at 25°C. One POD unit was defined as the change in 0.01 absorbance units per minute per gram FW.

Catalase (CAT) activity was determined using a commercial kit (Solarbio, Cat. No. BC0200) by monitoring H_2_O_2_ decomposition at 240 nm. The reaction was initiated by adding 0.3% (v/v) H_2_O_2_ to enzyme extract in 50 mM sodium phosphate buffer (pH 7.0), and the decrease in absorbance was recorded for 3 min at 25°C. One CAT unit was defined as the amount of enzyme decomposing 1 μmol H_2_O_2_ per minute.

#### 2.4.4 Malondialdehyde content

Malondialdehyde (MDA) content, an indicator of lipid peroxidation, was determined using a commercial kit (Solarbio, Cat. No. BC0020) by the thiobarbituric acid (TBA) method. Fresh leaf tissue (0.2 g) was homogenized in 5 mL of 10% (w/v) trichloroacetic acid and centrifuged at 10, 000 × g for 10 min. The supernatant was reacted with 0.5% (w/v) TBA in 20% TCA at 95°C for 30 min. After cooling and centrifugation, absorbance was measured at 532 nm and 600 nm. MDA content was calculated using an extinction coefficient of 155 mM^-1^·cm^-1^ and expressed as nmol·g^-1^ FW.

### 2.5 Transcriptome sequencing and analysis

Total RNA was extracted from frozen leaf samples (~100 mg) using TRIzol reagent (Invitrogen, Carlsbad, CA, USA, Cat. No. 15596018). RNA integrity was assessed by 1.2% agarose gel electrophoresis; concentration and purity were quantified using a NanoDrop 2000 spectrophotometer (Thermo Fisher Scientific, Waltham, MA, USA). Samples with A_260_/A_280_ ratios of 1.9-2.1, A_260_/A_230_ ratios > 2.0, and RNA integrity number (RIN) ≥ 7.0 (Agilent 2100 Bioanalyzer, Agilent Technologies, Santa Clara, CA, USA) were used for library construction.

Strand-specific cDNA libraries were constructed with the MGIEasy RNA Library Prep Kit (MGI Tech Co., Ltd., Shenzhen, China, Cat. No. 1000005250). mRNA was enriched using oligo(dT) magnetic beads, fragmented, and reverse-transcribed into cDNA. After end repair, A-tailing, and adapter ligation, fragments of 200–500 bp were size-selected and amplified by PCR. Libraries were sequenced on the DNBSEQ-T7 platform (MGI Tech) at BGI Genomics (Shenzhen, China) with paired-end reads (2 × 150 bp).

Raw reads were filtered to remove adapters, reads containing >10% unknown nucleotides (N), and low-quality reads (>50% bases with Phred Q ≤ 5). Clean reads were aligned to the *Rhododendron simsii* reference genome (GenBank assembly: GCA_014282245.1; BioSample: SAMN13241185) using HISAT2 (v2.2.1). The overall alignment rate was 77.60% (mapped reads), with 73.78% uniquely mapped reads. Gene expression was quantified as FPKM using StringTie (v2.1.7). Differential expression analysis was performed with DESeq2 (v1.38.0) in R (v4.2.1). Genes with |log_2_(fold change)| ≥ 1 and adjusted P-value (padj) < 0.05 were identified as differentially expressed genes (DEGs). Four pairwise comparisons were performed: LT vs CK, A vs CK, ALT vs LT, and ALT vs A. GO enrichment analysis was conducted using clusterProfiler (v4.6.0); KEGG pathway enrichment was performed using KOBAS (v3.0) with corrected *P*-value < 0.05 as the significance threshold.

To dissect the molecular mechanisms underlying ABA-mediated cold priming, DEGs were partitioned into five biologically defined subsets based on their differential expression patterns across comparisons: (i) “Pre-activated” genes—upregulated in A vs CK but not significantly changed in LT vs CK, representing ABA-primed genes that remain stable under cold stress; (ii) “ABA-unlocked cold-responsive” genes—significantly changed in ALT vs CK but not in LT vs CK, representing cold-responsive genes requiring ABA-primed background; (iii) “Damage-suppressed” genes—significantly changed in LT vs CK but attenuated in ALT vs CK, representing cold-induced dysregulation partially rescued by ABA; (iv) “Cold core” genes—significantly changed in both LT vs CK and ALT vs CK with the same direction, representing conserved ABA-independent cold responses; and (v) “ABA-specific” genes—significantly changed in A vs CK and ALT vs CK but not in LT vs CK, representing pure ABA signaling targets. Independent GO enrichment analyses were performed on each subset using clusterProfiler (v4.6.0) with corrected *P*-value < 0.05.

### 2.6 Proteome analysis

Total protein was extracted from frozen leaf samples (~300 mg) using SDT lysis buffer (4% SDS, 100 mM DTT, 150 mM Tris-HCl, pH 8.0). Samples were ground in liquid nitrogen, lysed at 95°C for 5 min, and centrifuged at 14, 000 × g for 15 min at 4°C. Protein concentration was determined using a BCA Protein Assay Kit (Thermo Fisher Scientific, Cat. No. 23225).

Proteome analysis was performed by Ultra-Fast quantitative proteomics. Samples were processed by the filter-aided sample preparation (FASP) method. Briefly, 200 μg protein was loaded onto a 10 kDa centrifugal filter unit (Sartorius, Göttingen, Germany, Cat. No. VS01T22), reduced with 50 mM DTT at 37°C for 30 min, alkylated with 100 mM IAA at room temperature for 30 min, and digested overnight at 37°C with sequencing-grade trypsin (Promega, Madison, WI, USA, Cat. No. V5113, enzyme-to-protein ratio 1:50, w/w). Peptides were desalted using C18 solid-phase extraction columns (Waters, Milford, MA, USA, Cat. No. WAT054960).

DIA analysis utilized the Vanquish Neo system(Thermo FisherScientific) for chromatographic separation. Samples separated by nano-flow high-performance liquid chromatography were subjected to DIA (Data-Independent Acquisition) mass spectrometry analysis using the Orbitrap Astral high-resolution mass spectrometer (Thermo Scientific). The detection mode was positive ion mode, with a precursorion scan range of 380-980m/z, a primary mass resolution of 240000 at 200 m/z, a Normalized AGC Target of 500%, and a Maximum IT of 5ms. MS2 was performed using DIA data acquisition mode, with 299 scan windows, an Isolation Window of 2 Th, HCD Collision Energy of 25%, a Normalized AGC Target of 500%, and a Maximum IT of 3 ms.

MS raw data were analyzed using DIA-NN(v1.8.1) with a library-free method. The pep.fa database (A total of 32, 999 sequences from the *R. simsii* genome) was used to create a spectral library with deep learning-based spectrum prediction. The match between runs (MBR) option was employed to generate a spectral library from data-independent acquisition (DIA) data, followed by reanalysis using this library. False discovery rate (FDR) of search results was adjusted to <1% at both protein and precursor ion levels. The remaining identifications were used for further quantification analysis. Protein quantification was performed using the MaxLFQ algorithm. DEPs were identified using the DEP R package (v1.18.0) with |log_2_(fold change)| ≥ 0.263 and adjusted *P*-value < 0.05.

### 2.7 Integrated transcriptome-proteome analysis

Integrated analysis of DEGs and DEPs was performed by matching gene identifiers across datasets. Pearson correlation coefficients were calculated for log_2_-transformed fold changes of corresponding mRNA-protein pairs. Nine-quadrant analysis was conducted to classify expression trends into nine categories based on the direction and significance of changes at both levels: Quadrant 1 - both up-regulated; Quadrant 2 - mRNA up, protein unchanged; Quadrant 3- mRNA up, protein down; Quadrant 4 - mRNA unchanged, protein up; Quadrant 5- both unchanged; Quadrant 6 - mRNA unchanged, protein down; Quadrant 7 - mRNA down, protein up; Quadrant 8 - mRNA down, protein unchanged; Quadrant 9 - both down-regulated. Joint KEGG pathway enrichment was performed by mapping both DEGs and DEPs onto KEGG pathways, and overlapping pathways were visualized using Venn diagrams.

### 2.8 qRT-PCR validation

To validate the transcriptome data, 15 DEGs with varying expression patterns were selected for quantitative real-time PCR (qRT-PCR) analysis.Total RNA was reverse-transcribed using the PrimeScript RT Reagent Kit (TaKaRa, Dalian, China). qRT-PCR was performed on a QuantStudio 3 Real-Time PCR System (Applied Biosystems) with SYBR Premix Ex Taq II (TaKaRa). *EF1-α* was used as the internal reference gene. Gene-specific primers were designed using Primer Premier 5.0 software and are listed in **Supplementary Table 1**. The 2^-ΔΔCt^ method was employed to calculate relative expression levels. Each reaction was performed with three biological replicates and three technical replicates, followed by melt curve analysis to ensure primer specificity.

### 2.9 Statistical analysis

All physiological data are presented as mean ± standard deviation (SD) of three biological replicates. Data were analyzed by one-way ANOVA followed by Duncan’s multiple range test using SPSS Statistics (v26.0, IBM Corp., Armonk, NY, USA). Significance was set at *P* < 0.05. For omics data, adjusted *P*-values were calculated using the Benjamini-Hochberg method. Figures were generated with GraphPad Prism (v9.0) and R (v4.2.1). Significance levels are denoted as follows: *P < 0.05, **P < 0.01, ***P < 0.001. ns, not significant (*P* ≥ 0.05).

## 3 Results

### 3.1 Effects of exogenous ABA on phenotype and photosynthetic pigments under cold stress

#### 3.1.1 Cold injury symptoms and phenotypic responses

**Figure 1:**
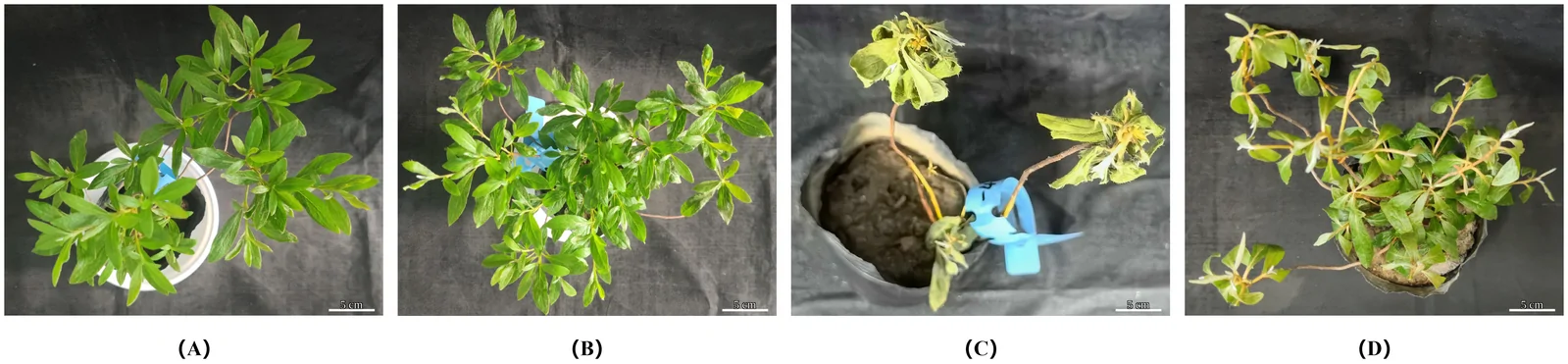
Phenotypic responses of *R. yedoense* var. *poukhanense* to exogenous ABA under normal and freezing temperatures after 5 days of treatment. **(A)** CK: 22 °C control; **(B)** A: 22 °C with 30 mg·L^-1^ ABA; **(C)** LT: -4 °C; **(D)** ALT: -4 °C with 30 mg·L^-1^ ABA. Scale bar = 5 cm, calibrated against the 15-cm diameter pot.

Visible cold injury symptoms were evaluated after 5 days of treatment to assess the protective efficacy of exogenous ABA on *R. yedoense* var. *poukhanense* cutting seedlings. As shown in **Figures 1A, B**, seedlings in the CK (22°C) and A (22°C + 30 mg·L^-1^ ABA) groups exhibited vigorous growth with fully expanded dark green leaves throughout the experimental period, confirming that exogenous ABA at 30 mg·L^-1^ exerted no phytotoxic effects under normal temperature.

Under severe freezing at -4°C, marked phenotypic divergence emerged between treatments. After 5 days, LT group seedlings displayed severe wilting, and massive leaf abscission (**Figure 1C**). By contrast, ALT group seedlings characterized by moderate leaf curling, and limited leaf abscission (**Figure 1D**). ABA pretreatment thus alleviated but could not fully prevent freezing-induced tissue damage.

#### 3.1.2 Photosynthetic pigment contents

The contents of Chl a, Chl b, xanthophyll, and carotenoid were significantly influenced by low-temperature stress and exogenously applied ABA (**Table 1**). Under normal temperature (22°C), no significant differences were detected between CK and A groups for any of the four pigments at 0 d or 5 d (*P* > 0.05), confirming that ABA alone does not affect baseline photosynthetic pigment levels under non-stress conditions.

**Table 1:** Effects of exogenous ABA on photosynthetic pigment contents in *R. yedoense* var. *poukhanense* leaves under cold stress (mg·g^-1^ FW).

Pigment	CK 0 d	CK 5 d	A 5 d	LT 5 d	ALT 5 d
Chl a	2.18 ± 0.14a	2.14 ± 0.12a	2.08 ± 0.15a	0.68 ± 0.09c	0.92 ± 0.11b
Chl b	0.64 ± 0.06a	0.62 ± 0.07a	0.59 ± 0.06a	0.15 ± 0.04c	0.22 ± 0.05b
Xanthophyll	0.30 ± 0.05a	0.29 ± 0.04a	0.28 ± 0.05a	0.08 ± 0.02c	0.12 ± 0.03b
Carotenoid	0.51 ± 0.07a	0.49 ± 0.06a	0.47 ± 0.06a	0.14 ± 0.03c	0.21 ± 0.04b

Following 5 days at -4°C, all four pigments exhibited dramatic depletion in the LT group. Chl a decreased to 0.68 ± 0.09 mg·g^-1^ FW, a 68.2% reduction versus CK 5 d (2.14 ± 0.12 mg·g^-1^ FW; *P* < 0.001). Chl b showed an even more pronounced decline, falling to 0.15 ± 0.04 mg·g^-1^ FW (75.8% decrease; *P* < 0.001), indicating greater sensitivity to freezing stress than Chl a, consistent with the lability of light-harvesting complex-associated pigments during chloroplast membrane disruption. Xanthophyll and carotenoid contents decreased by 72.4% and 71.4%, respectively, relative to CK 5 d (*P* < 0.001 for both).

These results demonstrate that temperature is the principal driver of pigment degradation, and that exogenous ABA confers partial protection at -4°C.

### 3.2 Effects of exogenous ABA on osmotic regulation substances

Osmotic regulation substances: soluble sugar (SS), soluble protein (SP), and free proline (Pro) were quantified to evaluate cellular osmoprotective responses (**Table 2**).

**Table 2:** Effects of exogenous ABA on osmotic regulation substance contents in *R. yedoense* var. *poukhanense* leaves under cold stress.

Osmolyte	CK 0 d	CK 5 d	A 5 d	LT 5 d	ALT 5 d
Soluble sugar (mg·g^-1^ FW)	8.52 ± 0.73b	8.48 ± 0.68b	21.79 ± 1.85c	38.10 ± 3.21d	40.40 ± 3.56d
Soluble protein (mg·g^-1^ FW)	12.35 ± 1.02b	12.18 ± 0.95b	39.62 ± 3.42c	36.68 ± 3.15c	25.73 ± 2.28d
Proline (μg·g^-1^ FW)	6.42 ± 0.58b	6.35 ± 0.52b	11.78 ± 1.05c	28.65 ± 2.48d	29.27 ± 2.61d

#### 3.2.1 Soluble sugar and soluble protein contents

Soluble sugar content remained stable under normal temperature. Freezing at -4°C for 5 days elicited dramatic SS accumulation in the LT group, reaching 38.10 ± 3.21 mg·g^-1^ FW, a 349.21% increase versus CK 5 d (*P* < 0.001). Exogenous ABA alone at normal temperature also promoted SS accumulation: the A 5 d group recorded 21.79 ± 1.85 mg·g^-1^ FW (+156.88% versus CK 5 d; *P* < 0.01). Under freezing stress, the ALT group exhibited a modest but significant further increase of 6.05% over LT 5 d (*P* < 0.05), reaching 40.40 ± 3.56 mg·g^-1^ FW. The near-saturation of SS accumulation in LT plants likely limits further ABA-mediated enhancement, as temperature-induced starch hydrolysis approaches maximal capacity. Thus, low temperature is the primary driver of SS accumulation with ABA providing quantitatively limited additive enhancement.

Soluble protein content followed a more complex pattern. Under normal temperature, SP remained stable between CK 0 d and CK 5 d. Low-temperature exposure induced a 200.97% increase in the LT group (36.68 ± 3.15 mg·g^-1^ FW; *P* < 0.001), reflecting synthesis of stress-related proteins including dehydrins and LEA proteins. ABA at normal temperature induced an even greater increase of 225.23% (39.62 ± 3.42 mg·g^-1^ FW; *P* < 0.001). Notably, under freezing stress, the ALT group exhibited SP content of 25.73 ± 2.28 mg·g^-1^ FW, which was 29.84% lower than the LT group (*P* < 0.01).

#### 3.2.2 Free proline content

Free proline exhibited massive accumulation under freezing stress. The LT group reached 28.65 ± 2.48 μg·g^-1^ FW after 5 days at -4°C, a 351.4% increase versus CK 5 d (*P* < 0.001). ABA at normal temperature significantly increased proline by 85.47% (11.78 ± 1.05 μg·g^-1^ FW; *P* < 0.01). Under freezing stress, the ALT group (29.27 ± 2.61 μg·g^-1^ FW) showed only a non-significant 2.15% increase over the LT group (*P* > 0.05), suggesting that proline biosynthetic capacity may already be saturated by severe cold or that proline is redistributed to other organs under combined stress.

In summary, all three osmolytes accumulated substantially under -4°C stress (200-350% increases). ABA promoted accumulation of all three osmolytes at normal temperature, but its additive effect under freezing varied by substance: positive for SS (+6.05%), negative for SP (-29.84%), and non-significant for proline (+2.15%).

### 3.3 Effects of exogenous ABA on antioxidant enzyme activities and MDA content

Low-temperature stress disrupts cellular redox homeostasis by enhancing ROS production. The activities of SOD, POD, CAT, and MDA content were evaluated to characterize ABA-mediated antioxidant protection (**Table 3**).

**Table 3:** Effects of exogenous ABA on antioxidant enzyme activities and MDA content in *R. yedoense* var. *poukhanense* leaves under cold stress.

Parameter	CK 0 d	CK 5 d	A 5 d	LT 5 d	ALT 5 d
SOD (U·g^-1^ FW)	168.5 ± 15.1a	166.0 ± 14.2a	204.6 ± 18.3b	92.0 ± 8.5d	120.6 ± 10.8c
POD (U·g^-1^ FW)	108.2 ± 9.8a	106.3 ± 9.1a	115.9 ± 10.2b	56.8 ± 5.4d	68.2 ± 6.3c
CAT (U·g^-1^ FW)	41.8 ± 3.9a	40.9 ± 3.6a	53.8 ± 4.9b	15.1 ± 1.8d	21.7 ± 2.3c
MDA (nmol·g^-1^ FW)	8.15 ± 0.72b	8.42 ± 0.78b	7.68 ± 0.65b	24.42 ± 2.18d	17.41 ± 1.56c

#### 3.3.1 SOD and POD activities

SOD activity was effectively induced by ABA at normal temperature: the A 5 d group exhibited 204.6 ± 18.3 U·g^-1^ FW, a 23.3% increase versus CK 5 d (166.0 ± 14.2 U·g^-1^ FW; *P* < 0.01). At -4°C, LT group SOD activity declined sharply to 92.0 ± 8.5 U·g^-1^ FW (-44.6%; *P* < 0.001). In the ALT group, SOD recovered to 120.6 ± 10.8 U·g^-1^ FW (+31.1% versus LT; *P* < 0.01) but remained 27.3% below the CK 5 d baseline.

POD activity exhibited a broadly similar pattern. The A 5 d group showed 115.9 ± 10.2 U·g^-1^ FW (+9.0% versus CK 5 d; *P* < 0.05). After 5 days at -4°C, LT group POD decreased to 56.8 ± 5.4 U·g^-1^ FW (−46.6%; *P* < 0.001). ALT group POD recovered to 68.2 ± 6.3 U·g^-1^ FW (+20.0% versus LT; *P* < 0.05) but remained 35.8% below the CK 5 d level.

#### 3.3.2 CAT activity

CAT displayed the most dramatic response among the three antioxidant enzymes. Under normal temperature, ABA induced the strongest enhancement: A 5 d CAT was 53.8 ± 4.9 U·g^-1^ FW, a 31.6% increase over CK 5 d (40.9 ± 3.6 U·g^-1^ FW; *P* < 0.01). At -4°C, LT group CAT plummeted to 15.1 ± 1.8 U·g^-1^ FW (-63.1%; *P* < 0.001), the most severe inhibition observed. Notably, ALT group CAT recovered to 21.7 ± 2.3 U·g^-1^ FW, representing a 43.6% increase versus LT (*P* < 0.01) - the largest relative recovery among the three enzymes. This preferential CAT recovery may reflect ABA-specific signaling to peroxisomal antioxidant pathways; CAT catalyzes H_2_O_2_ dismutation without consuming reducing equivalents, making it energetically advantageous when NADPH availability is limiting under freezing stress. Recovered activity remained 46.9% below baseline, highlighting the quantitative limits of ABA-mediated protection at -4°C.”

#### 3.3.3 MDA content

MDA content, a biomarker of membrane lipid peroxidation, increased dramatically under freezing stress. The LT group reached 24.42 ± 2.18 nmol·g^-1^ FW after 5 days- approximately 2.9-fold the CK 5 d level (8.42 ± 0.78 nmol·g^-1^ FW; *P* < 0.001). Notably, A 5 d MDA (7.68 ± 0.65 nmol·g^-1^ FW) was slightly lower than CK, suggesting ABA reduces baseline oxidative damage even without stress. Under freezing stress, ALT group MDA (17.41 ± 1.56 nmol·g^-1^ FW) was 28.7% lower than LT (*P* < 0.01), consistent with partially recovered antioxidant enzyme activities. However, ALT MDA remained approximately 2.1-fold higher than CK (*P* < 0.001), confirming that despite significant alleviation, ABA-pretreated seedlings still experienced considerable oxidative membrane damage under severe freezing conditions. The persistence of elevated MDA in the ALT group aligns with the incomplete recovery of the three antioxidant enzymes and further underscores that -4°C imposes dominant physiological constraints that ABA cannot fully override.

### 3.4 Transcriptome analysis

#### 3.4.1 Overview of DEGs across comparisons

**Figure 2:**
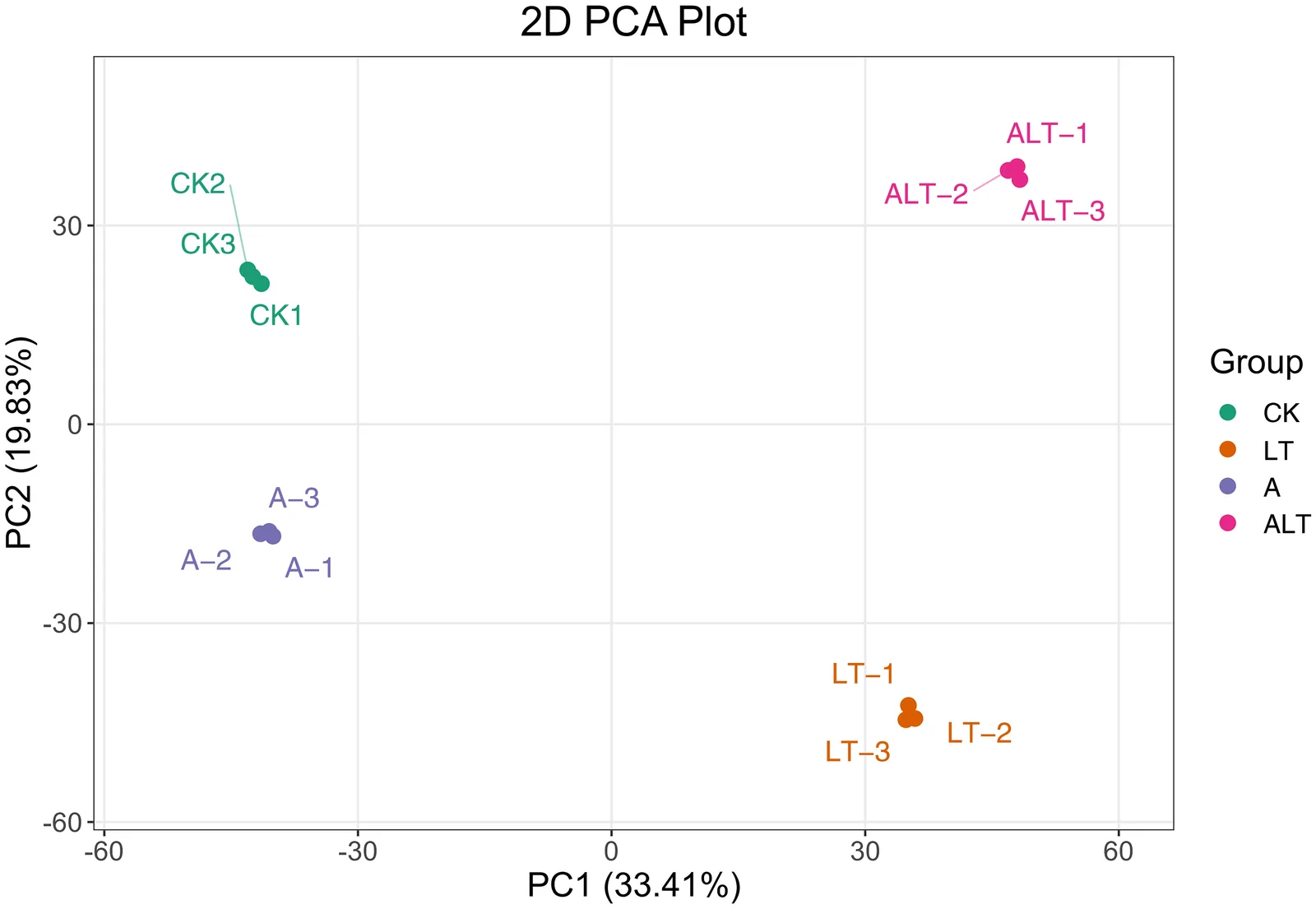
PCA of transcriptome data from *R. yedoense* var. *poukhanense* under four treatments.

RNA-seq generated a total of 35, 238 expressed genes across all 12 samples (three biological replicates per treatment). Principal component analysis (PCA) based on the global expression profile clearly separated the four treatment groups along PC1 and PC2, with biological replicates clustering tightly within each group, confirming high data reliability and reproducibility (**Figure 2**). PC1 (explaining the largest proportion of variance) primarily distinguished cold-treated samples (LT, ALT) from control samples (CK, A), while PC2 separated ABA-treated samples (A, ALT) from non-ABA samples (CK, LT), indicating that both cold stress and ABA treatment induced distinct and substantial transcriptional reprogramming.

DEG analysis using DESeq2 (|log_2_FC| >= 1, adjusted *P*-value < 0.05) revealed dramatic treatment-induced transcriptional changes (**Table 4**). The LT vs CK comparison showed the most extensive DEG set with 8, 444 differentially expressed genes (DEGs) (5, 857 upregulated, 2, 587 downregulated), reflecting the severe transcriptional impact of -4 C freezing stress. ABA pretreatment alone (A vs CK) induced 4, 637 DEGs (2, 500 up, 2, 137 down), indicating that exogenous ABA alone triggers substantial transcriptional reprogramming even under normal temperature. The ALT vs CK comparison identified 6, 481 DEGs (4, 321 up, 2, 160 down), representing a 23% reduction relative to LT vs CK (8, 444), indicating that ABA pretreatment attenuated the overall scope of cold-induced transcriptional reprogramming. Notably, the ALT vs LT comparison yielded only 1, 690 DEGs (897 up, 793 down), representing genes specifically modulated by ABA under freezing conditions beyond the established cold response. The modest number of ALT vs LT-specific DEGs suggests that most cold-responsive pathways were pre-activated during the ABA priming phase at 22 °C, with minimal additional transcriptional perturbation required upon transfer to -4°C—consistent with a priming rather than a *de novo* protective response. Similarly, ALT vs A comparison revealed 6, 102 DEGs (3, 767 up, 2, 335 down), further supporting the protective priming role of ABA under subsequent cold stress.

**Table 4:** Statistics of DEGs across five comparisons.

Comparison	Upregulated	Downregulated	Total
LT vs CK	5, 857	2, 587	8, 444
A vs CK	2, 500	2, 137	4, 637
ALT vs CK	4, 321	2, 160	6, 481
ALT vs LT	897	793	1, 690
ALT vs A	3, 767	2, 335	6, 102

**Figure 3:**
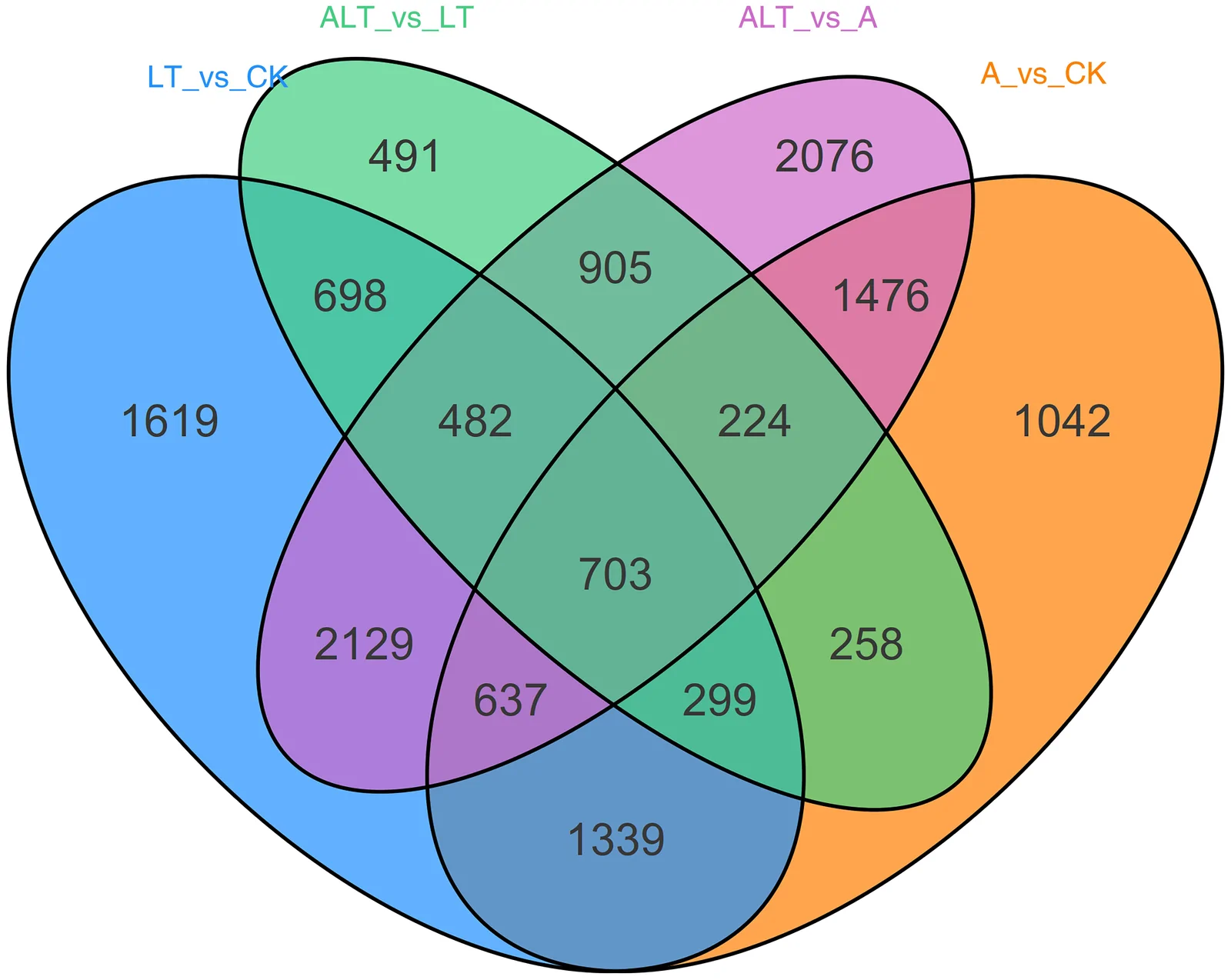
Venn diagram analysis of differentially expressed genes (DEGs) across four pairwise comparisons in *R. yedoense* var. *poukhanense*.

Venn diagram analysis of DEGs among the four pairwise comparisons (LT vs CK, A vs CK, ALT vs LT, and ALT vs A) revealed both shared and treatment-specific transcriptional responses (**Figure 3**). A core set of 637 DEGs was shared across all four comparisons, representing the common stress-responsive transcriptome activated by both low temperature and ABA signaling. This core set likely encompasses fundamental stress-adaptive genes including antioxidant enzymes, molecular chaperones, and osmolyte biosynthesis genes that respond independently of the specific stress modality.

#### 3.4.2 GO enrichment analysis

**Figure 4:**
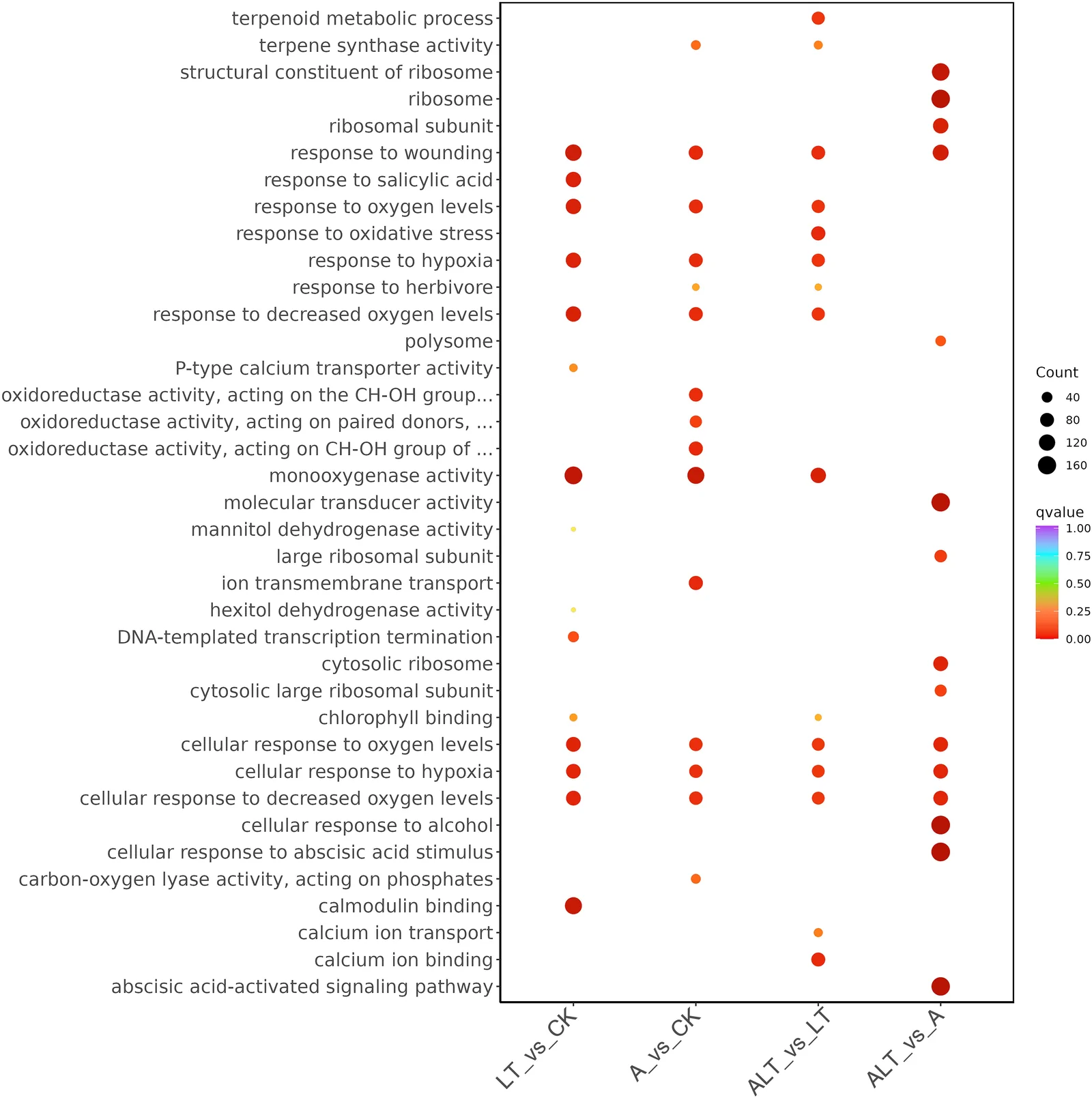
GO enrichment analysis of differentially expressed genes across four pairwise comparisons. The x-axis represents the comparison groups, the y-axis represents the enriched pathways, and the size of the points indicates the number of differentially expressed genes enriched in each pathway-larger points signify a greater number of enriched differentially expressed genes. The color of the points reflects the significance level of enrichment, with yellow (moderate significance) to dark red (high significance).

GO enrichment analysis was performed independently for all four pairwise comparisonss (LT vs CK, A vs CK, ALT vs LT, ALT vs A). The top enriched GO terms across comparisons are visualized as a bubble plot, where dot size represents the number of enriched DEGs and color intensity indicates the q-value significance (**Figure 4**).

The oxidative stress response cluster-encompassing “response to oxidative stress, “ “response to oxygen levels, “ “response to hypoxia, “ and “response to decreased oxygen levels”-was the most consistently enriched module across all comparisons, establishing oxidative stress defense as the core invariant component of both cold and ABA responses. LT vs CK exhibited the broadest spectrum, additionally enriching terpenoid metabolic process, multiple oxidoreductase activities, and calmodulin binding, consistent with severe freezing-induced oxidative damage and Ca²^+^ signal transduction. A vs CK showed a narrower overlapping profile with unique ion transmembrane transport enrichment but absence of terpenoid metabolism, indicating ABA alone primes redox capacity without triggering secondary metabolite accumulation. ALT vs LT preserved core oxidative responses while gaining chlorophyll binding and calcium ion transport, suggesting ABA pretreatment enhances photosynthetic protection under freezing. Notably, ALT vs A was uniquely dominated by ribosome-related terms (structural constituent of ribosome, ribosomal subunit, cytosolic ribosome) alongside abscisic acid-activated signaling pathway, indicating that low temperature in the ABA-primed background triggers extensive translational machinery remodeling. These patterns support a model wherein ABA priming establishes redox and translational preparedness, while cold drives oxidative defense and secondary metabolism, with their combination producing a functionally integrated stress response program.

#### 3.4.3 KEGG pathway enrichment analysis

**Figure 5:**
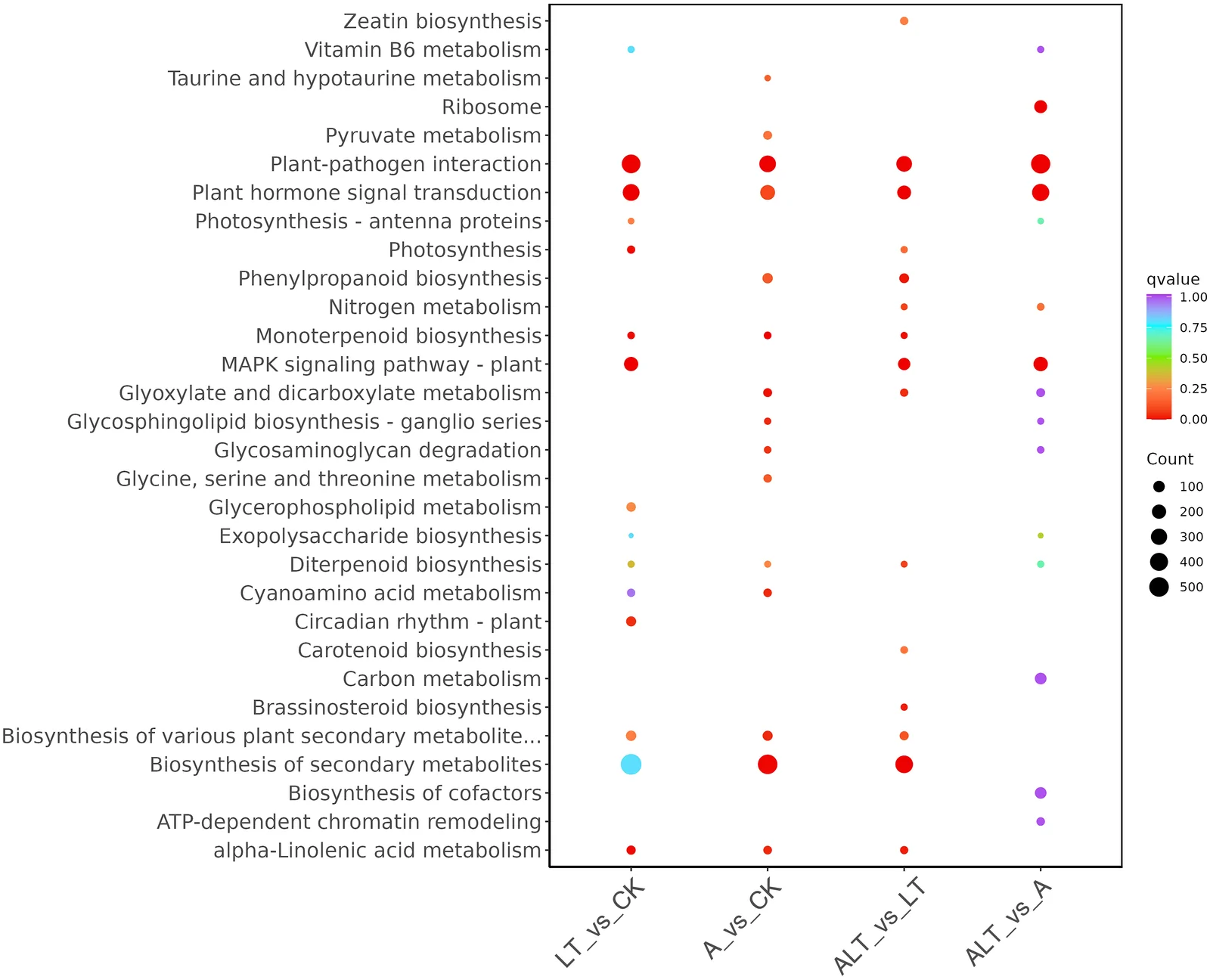
KEGG pathway enrichment analysis of differentially expressed genes across four pairwise comparisons. The x-axis represents the comparison groups, the y-axis represents the enriched pathways, and the size of the points indicates the number of differentially expressed genes enriched in each pathway-larger points signify a greater number of enriched differentially expressed genes. The color of the points reflects the significance level of enrichment, with redder colors indicating more significant enrichment.

KEGG pathway enrichment analysis across four pairwise comparisons revealed both conserved and treatment-specific metabolic and signaling signatures (**Figure 5**). Plant-pathogen interaction, biosynthesis of secondary metabolites, monoterpenoid biosynthesis, and alpha-linolenic acid metabolism were consistently enriched across all four comparisons, establishing these pathways as core components of both cold and ABA responses. MAPK signaling pathway - plant and plant hormone signal transduction were prominently enriched in LT vs CK, ALT vs LT, and ALT vs A, but attenuated in A vs CK, indicating that MAPK-mediated signal transduction is primarily cold-driven rather than ABA-responsive. Photosynthesis and photosynthesis - antenna proteins were specific to LT vs CK, consistent with cold-induced photodamage, while ribosome emerged as a dominant enrichment in A vs CK and ALT vs A, indicating ABA-priming and combined stress both trigger substantial translational machinery remodeling. ALT vs LT uniquely enriched zeatin biosynthesis and brassinosteroid biosynthesis, suggesting ABA buffering under freezing involves cytokinin and brassinosteroid cross-talk. Notably, ALT vs A displayed the broadest pathway spectrum, additionally enriching carbon metabolism, glycerophospholipid metabolism, and ATP-dependent chromatin remodeling, supporting that low temperature in the ABA-primed background activates extensive metabolic reprogramming and epigenetic modulation. Collectively, these patterns indicate that cold stress primarily drives MAPK signaling, photosynthetic impairment, and secondary metabolism, while ABA priming establishes ribosomal preparedness; their combination produces a metabolically integrated response through carbon metabolism, phospholipid remodeling, and hormone cross-talk.

#### 3.4.4 Transcription factor analysis

**Figure 6:**
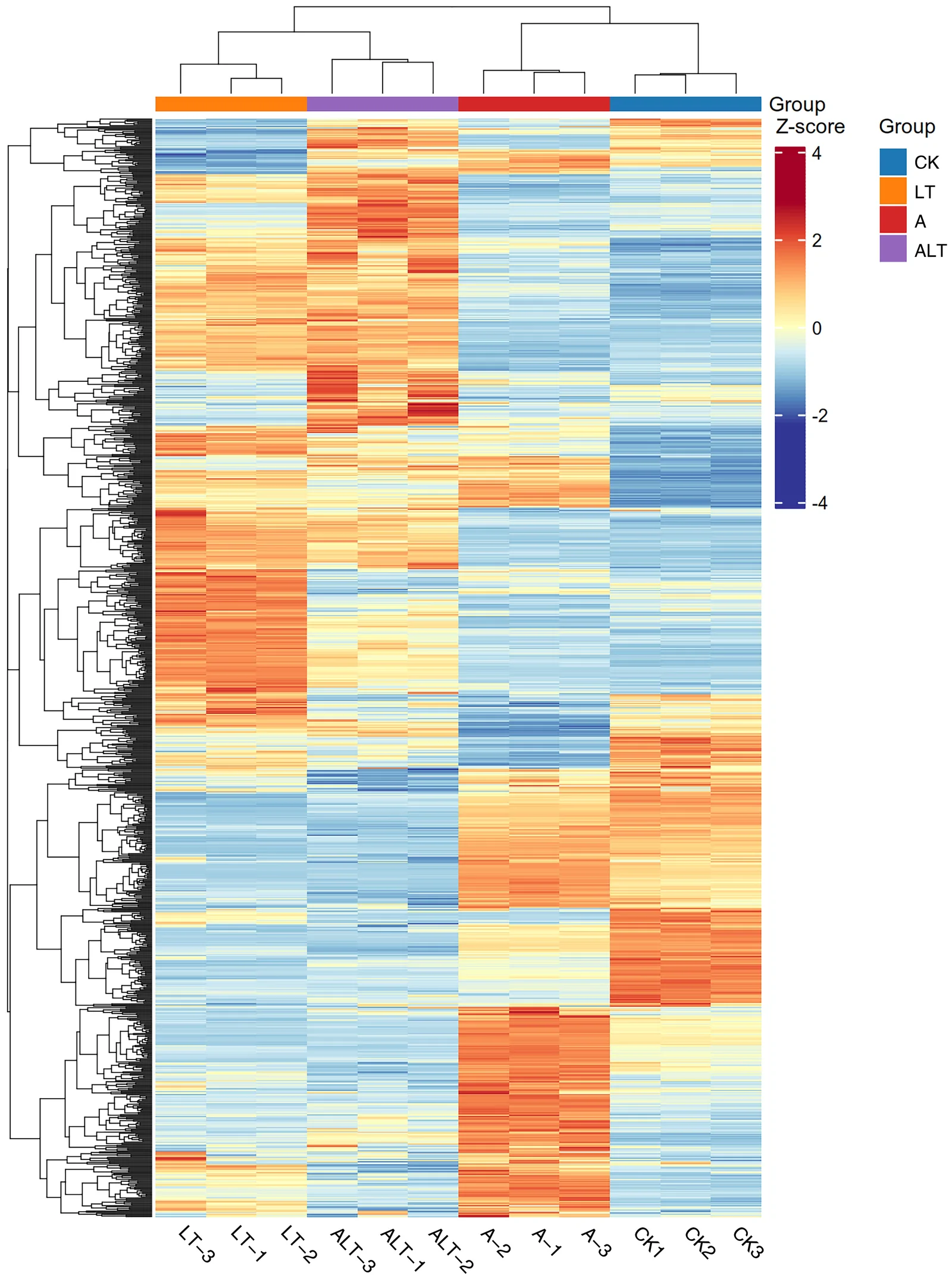
Hierarchical clustering heatmap of differentially expressed TFs across four treatments in *R. yedoense* var. *poukhanense*. The x-axis represents the sample names and the hierarchical clustering results, while the y-axis represents the differentially expressed transcription factors and the hierarchical clustering results. Red indicates high expression, and blue indicates low expression.

A total of 1, 002 transcription factors (TFs) belonging to 43 families were identified across the transcriptome. Hierarchical clustering of TF expression profiles revealed two major sample clusters: CK and A samples grouped together, while LT and ALT samples formed a separate cluster, indicating that low temperature is the primary driver of TF reprogramming, with ABA alone exerting relatively modest effects on the global TF landscape (**Figure 6**).

Among all pairwise comparisons, LT vs CK induced the most extensive TF reprogramming with 416 upregulated and 280 downregulated TFs (696 total), followed closely by ALT vs CK (411 up, 280 down, 691 total), while A vs CK showed the smallest response (303 up, 171 down, 474 total). This pattern confirms that ABA pretreatment does not substantially reduce the number of cold-responsive TFs at the transcriptome level, but rather modulates their expression amplitude-consistent with the ABA priming effect. The AP2/ERF-ERF family was the largest group (84 members), with DREB1A/CBF3, DREB1B/CBF1, and DREB1C/CBF2 all strongly upregulated under LT and maintained in ALT, establishing the CBF cold regulon as a core conserved module. The MYB family (59 members) and WRKY family (47 members) showed divergent patterns: WRKY40 and MYB2 peaked under LT but were attenuated in ALT, whereas WRKY53 and MYB44 maintained moderate induction across both cold conditions. The bZIP family (30 members), including ABF2 and ABF3, was uniquely enriched in A vs CK and ALT vs A comparisons, highlighting ABA-specific signaling activation. Notably, the NAC family (36 members) and C2H2 family (35 members) were prominent in LT vs CK but substantially buffered in ALT vs LT, suggesting that ABA pretreatment specifically attenuates ROS-activated TF modules while preserving core cold-regulon function. Collectively, these TF dynamics reveal that ABA-mediated cold priming operates through selective amplification of CBF/ABF pathways and dampening of WRKY/NAC/C2H2 oxidative stress modules, rather than global suppression of cold-responsive transcription.

#### 3.4.5 Targeted enrichment analysis of ABA priming-associated gene subsets

To move beyond generic pathway enrichment and dissect the molecular basis of ABA-mediated cold priming, DEGs were partitioned into five biologically defined subsets based on their differential expression patterns across comparisons. Independent GO enrichment analyses were performed on each subset (**Table 5**).

**Table 5:** GO enrichment summary of ABA priming-associated gene subsets.

Subset	Definition	Total genes	Genes with GO	Significant GO terms	Key enriched functions
Pre-activated	Up in A vs CK; stable in LT vs CK	1, 397	1, 076	45	Nucleotide/ATP binding, ribonucleoside phosphate binding (energy priming)
ABA unlocked cold-responsive	Sig in ALT vs CK; not sig in LT vs CK	2, 224	1, 758	11	Cytosolic ribosome, carboxylic acid metabolism (translational enablement)
Damage-suppressed	Sig in LT vs CK; attenuated in ALT vs CK	5, 619	4, 383	12	Chloroplast thylakoid, photosynthesis, electron transport chain (photoprotection)
Cold core	Sig in both LT vs CK and ALT vs CK	4, 114	3, 284	6	Response to chemical, hormone, organic substance (conserved cold response)
ABA-specific	Sig in A vs CK and ALT vs CK; not sig in LT vs CK	483	399	0	Insufficient statistical power

The “Pre-activated” subset (1, 397 genes upregulated by ABA at 22°C but stable under cold stress) showed the strongest enrichment signal (45 significant GO terms), with prominent over-representation of nucleotide binding, ATP binding, and ribonucleoside phosphate binding activities. These findings indicate that ABA pretreatment establishes an energy-primed state, preparing the cellular ATP-generating and nucleotide-metabolizing machinery for subsequent cold challenge (**Supplementary Figure 2A**).

The “ABA-unlocked cold-responsive” subset (2, 224 genes responsive to cold only in the ABA-primed background) was enriched in mitotic cell cycle, cytosolic ribosome, and carboxylic acid metabolic processes. This suggests that ABA priming enables cold-induced translational reprogramming and metabolic reconfiguration that cannot occur in non-primed plants (**Supplementary Figure 2B**).

The “Damage-suppressed” subset (5, 619 genes whose cold-induced dysregulation was attenuated by ABA) was overwhelmingly dominated by photosynthesis-related GO terms, including chloroplast thylakoid membrane, photosynthesis, and photosynthetic electron transport chain. This confirms that ABA pretreatment specifically protects the photosynthetic apparatus from freezing-induced photodamage, consistent with the partial pigment preservation observed at the physiological level (**Supplementary Figure 2C**).

The “Cold core” subset (4, 114 genes responding to cold regardless of ABA status) was enriched in general stress response terms including response to chemical stimulus (including cellular response to chemical stimulus), response to organic substance, and response to hormone, representing the conserved, ABA-independent freezing tolerance machinery (**Supplementary Figure 2D**).

The “ABA-specific” subset (483 genes responsive to ABA independent of cold stress) did not yield significant GO enrichment, likely due to limited statistical power from the small gene set. Collectively, these subset-specific enrichment patterns demonstrate that ABA priming operates through distinct functional modules—energy priming, translational enablement, and photoprotection—rather than through global suppression of cold-responsive transcription.

### 3.5 Proteome analysis

#### 3.5.1 Overview of differentially expressed proteins

Data-independent acquisition mass spectrometry (DIA-MS) identified a total of 5, 688 proteins across all 12 samples with high quantitative reproducibility (median coefficient of variation < 15% across technical replicates). Principal component analysis of the proteome data showed clear separation of cold-treated samples (LT, ALT) from control samples (CK, A) along PC1, with ABA-treated samples (A, ALT) partially overlapping, indicating that cold stress induced more pronounced proteome-level changes than ABA treatment alone.

Differential expression analysis (|log_2_FC| >= 0.263, adjusted P-value < 0.05) revealed 1, 244 differentially expressed proteins (DEPs) (468 upregulated, 776 downregulated) in LT vs CK, 597 DEPs (279 up, 318 down) in A vs CK, 1, 461 DEPs (644 up, 817 down) in ALT vs CK, 534 DEPs (310 up, 224 down) in ALT vs LT, and 1, 580 DEPs (723 up, 857 down) in ALT vs A (**Table 6**). Notably, the LT vs CK comparison showed a marked excess of downregulated DEPs (776 down vs. 468 up), contrasting with the transcriptome where upregulated DEGs predominated (5, 857 up vs. 2, 587 down).

**Table 6:** Statistics of DEPs across five comparisons.

Comparison	Upregulated	Downregulated	Total
LT vs CK	468	776	1, 244
A vs CK	279	318	597
ALT vs CK	644	817	1, 461
ALT vs LT	310	224	534
ALT vs A	723	857	1, 580

**Figure 7:**
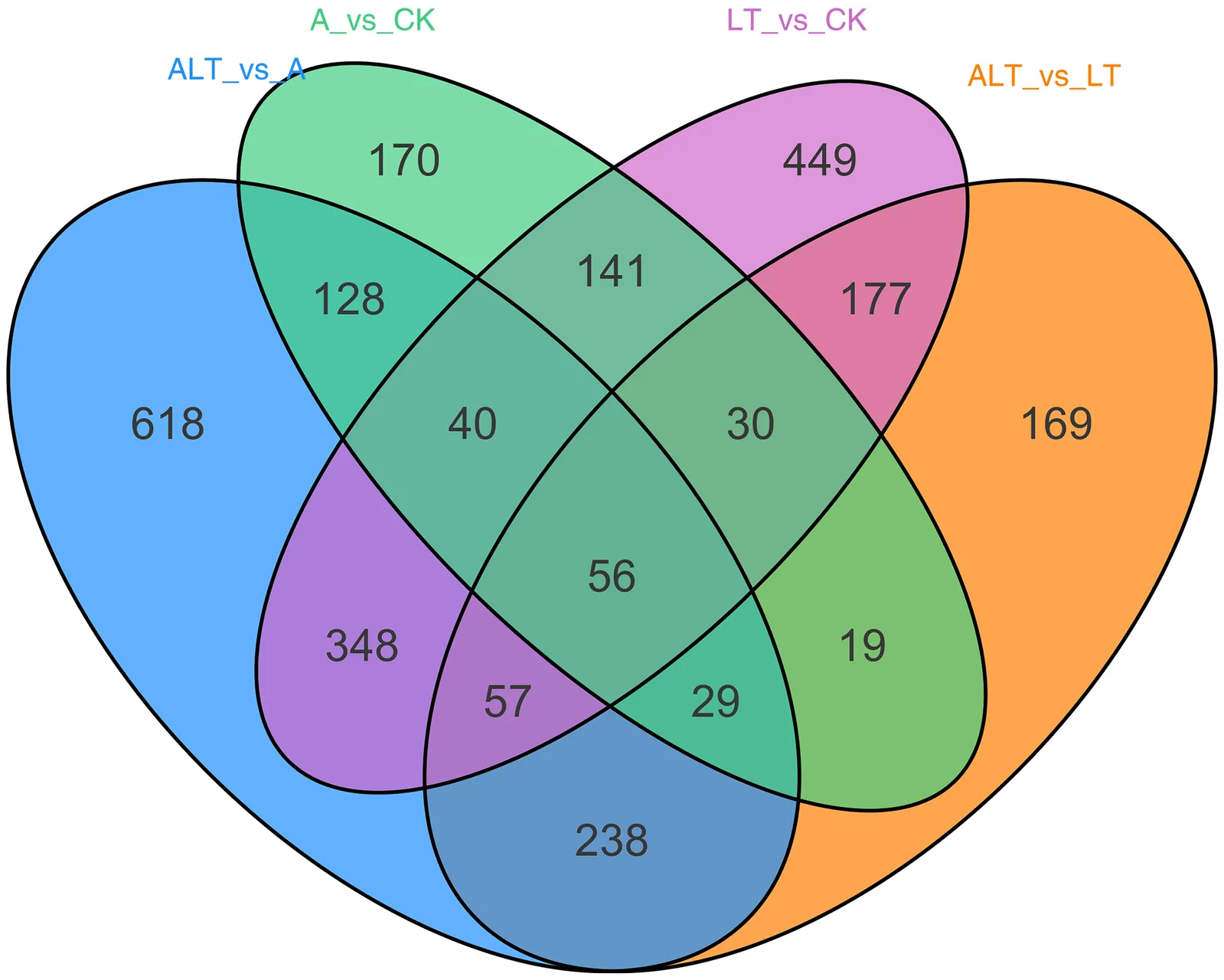
Venn diagram of DEPs across four pairwise comparisons in *R. yedoense* var. *poukhanense*.

Venn diagram analysis of DEPs across four pairwise comparisons (LT vs CK, A vs CK, ALT vs LT, ALT vs A) revealed substantial differences in proteome remodeling magnitude and treatment specificity (**Figure 7**). The ALT vs LT comparison produced the largest unique DEP set (618 proteins), representing proteins whose abundance changed specifically when ABA was added to cold-stressed seedlings-consistent with ABA’s role in modulating pre-established cold responses. LT vs CK yielded 449 unique DEPs, representing the core cold-responsive proteome independent of ABA priming. In contrast, both A vs CK and ALT vs A showed substantially smaller unique DEP sets (170 and 169, respectively), confirming that ABA alone-whether at normal or subzero temperature-drives relatively modest proteomic changes compared to cold stress. A core set of 56 DEPs was shared across all four comparisons, encompassing proteins universally responsive to both temperature change and ABA signaling. Notably, the combined total of DEPs involving cold treatment (LT vs CK: 449 + ALT vs LT: 618 = 1, 067) far exceeded those associated with ABA treatment (A vs CK: 170 + ALT vs A: 169 = 339), quantitatively confirming that low temperature is the dominant driver of proteome remodeling, while ABA primarily acts as a modulator of cold-established protein networks.

#### 3.5.2 GO enrichment analysis

GO enrichment analysis was performed independently for all four pairwise comparisons (LT vs CK, A vs CK, ALT vs LT, ALT vs A), revealing treatment-specific and shared functional signatures at the protein level (**Supplementary Figure 3**).

LT vs CK exhibited the broadest GO enrichment spectrum (224 terms), with “translation” (64 DEPs, 87.5% downregulated), “ribosome” (48 DEPs, 87.5% downregulated), and “structural constituent of ribosome” (64 DEPs, 89% downregulated) as the most prominent categories, indicating that -4°C freezing severely suppresses translational machinery and ribosomal protein abundance. “Chloroplast” (36 DEPs, 61% downregulated) and “oxidoreductase activity” (32 DEPs) were also enriched, reflecting cold-induced photodamage and redox imbalance. A vs CK showed a narrower profile (184 terms) with a similar pattern of ribosomal suppression-”translation” (31 DEPs, 94% downregulated) and “ribosome” (26 DEPs, 96% downregulated)-but additionally enriched “defense response” (19 DEPs) and “heme binding” (22 DEPs), suggesting that ABA alone at normal temperature modestly suppresses translation while activating defense-related proteins. Strikingly, ALT vs LT revealed a complete reversal of the ribosomal trend: “translation” (86 DEPs, 99% upregulated), “ribosome” (71 DEPs, 99% upregulated), and “structural constituent of ribosome” (87 DEPs, 100% upregulated) were the top enriched terms with near-unanimous upregulation and highly significant FDR values, demonstrating that ABA pretreatment under freezing conditions substantially restores translational capacity. ALT vs A displayed the most diverse enrichment profile (226 terms), with “translation” (81 DEPs, 93% upregulated) and “proteolysis” (52 DEPs, 62% upregulated) as top categories, alongside “carbohydrate metabolic process” (42 DEPs), “photosynthesis” (19 DEPs), and “chloroplast thylakoid membrane” (19 DEPs), indicating that low temperature in the ABA-primed background triggers extensive metabolic reprogramming, protein turnover, and partial photosynthetic recovery.

Collectively, these patterns reveal that cold stress dominantly suppresses ribosomal and translational proteins, ABA alone exerts a milder translational inhibition with defense priming, and ABA pretreatment under freezing dramatically restores translation while activating proteolysis and carbohydrate metabolism-a functional reprogramming profile distinct from either treatment alone.

#### 3.5.3 KEGG pathway enrichment analysis

KEGG pathway enrichment analysis across four pairwise comparisons revealed both conserved and treatment-specific metabolic signatures at the protein level (**Supplementary Figure 4**).

LT vs CK exhibited the broadest pathway spectrum (134 pathways), with “ribosome” as the top enriched pathway (70 DEPs, 84% downregulated), followed by “carbon metabolism” (56 DEPs, 68% downregulated), “biosynthesis of amino acids” (46 DEPs), and “protein processing in endoplasmic reticulum” (35 DEPs), collectively indicating that -4°C freezing severely suppresses translational machinery, central carbon metabolism, and protein quality control. A vs CK showed a narrower profile (117 pathways) with “ribosome” still dominating (31 DEPs, 87% downregulated), but uniquely enriching “phenylpropanoid biosynthesis” (22 DEPs), “MAPK signaling pathway – plant” (14 DEPs), and “plant-pathogen interaction” (14 DEPs), suggesting that ABA alone at normal temperature modestly suppresses translation while activating phenylpropanoid-mediated defense signaling. Strikingly, ALT vs LT revealed a complete reversal of the ribosomal trend: “ribosome” was the overwhelmingly dominant pathway (93 DEPs, 100% upregulated), accompanied by “spliceosome” (28 DEPs), “flavonoid biosynthesis” (13 DEPs), and “phenylpropanoid biosynthesis” (14 DEPs), demonstrating that ABA pretreatment under freezing conditions not only restores ribosomal protein abundance but also enhances secondary metabolite biosynthesis. ALT vs A displayed the most diverse metabolic reprogramming (133 pathways), with “ribosome” (90 DEPs, 91% upregulated), “carbon metabolism” (61 DEPs, 85% downregulated), “spliceosome” (45 DEPs), and “pyruvate metabolism” (28 DEPs, 89% downregulated) as top categories, indicating that low temperature in the ABA-primed background triggers extensive translational recovery coupled with redirection of carbon and pyruvate metabolism.

Collectively, these KEGG patterns reveal a coherent functional architecture: cold stress dominantly suppresses ribosomal and carbon metabolism proteins, ABA alone exerts milder translational inhibition with defense pathway priming, and ABA pretreatment under freezing dramatically restores translation and spliceosomal function while redirecting central carbon metabolism-a metabolic reprogramming profile that aligns precisely with the GO-derived observations.

### 3.6 Integrated transcriptome-proteome analysis

#### 3.6.1 Joint PCA and expression correlation

**Figure 8:**
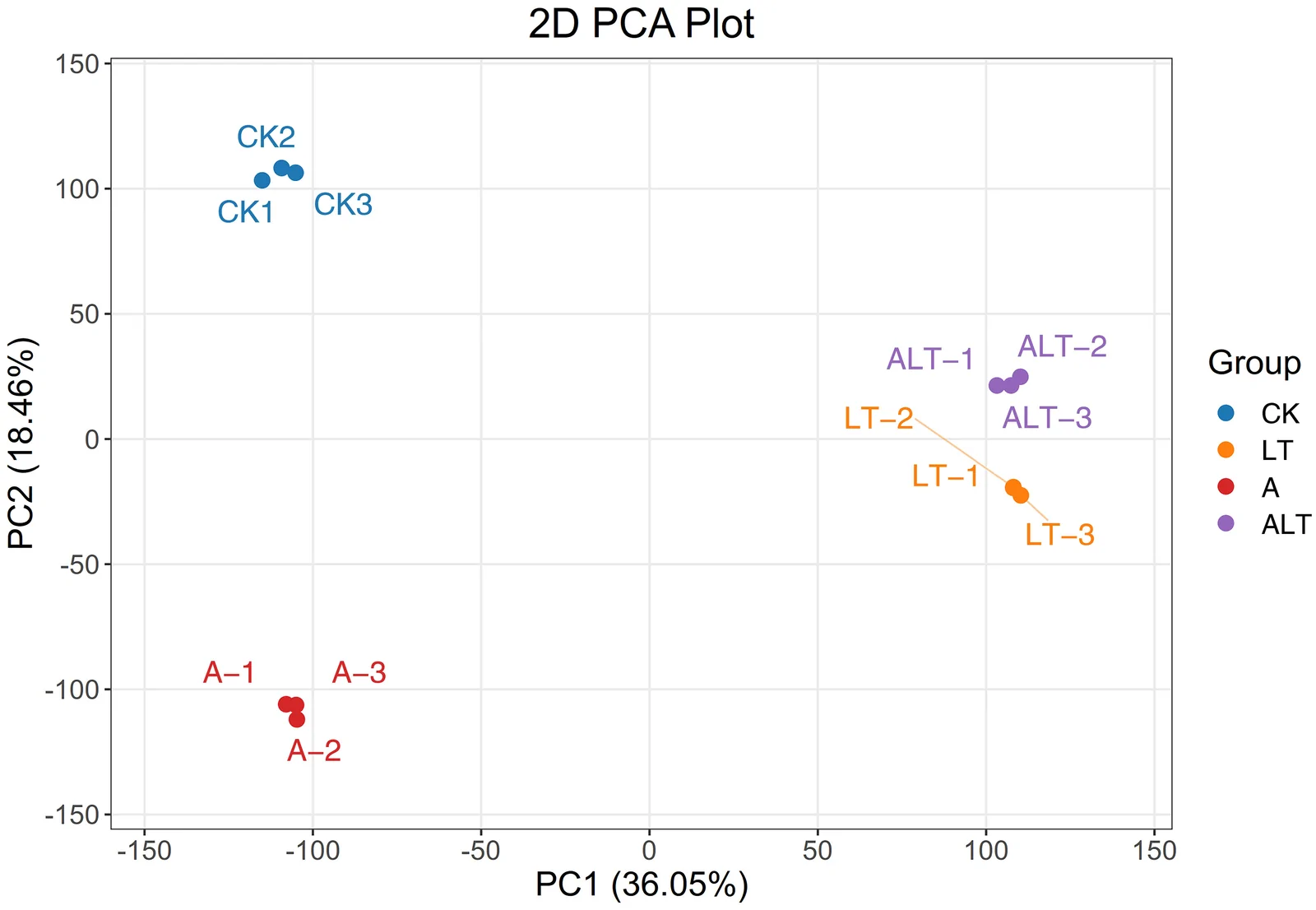
PCA of proteome data from *R. yedoense* var. *poukhanense* under four treatments.

Principal component analysis was performed on the combined transcriptome-proteome expression matrix to assess the overall concordance between mRNA and protein expression patterns (**Figures 2**, **8**). PC1 explained 36.0% of the total variance in the transcriptome (33.4% in the proteome) and primarily separated cold-treated samples (LT, ALT) from non-cold samples (CK, A), while PC2 explained 18.5% of the variance in the transcriptome (19.8% in the proteome) and distinguished ABA-treated from non-ABA samples. Notably, transcriptome and proteome samples from the same treatment group clustered adjacently but not identically, indicating partially correlated but distinct expression patterns between the two omics layers. The ALT samples showed the closest transcriptome-proteome concordance, suggesting that ABA pretreatment enhances the fidelity of transcription-to-translation coupling under cold stress.

**Figure 9:**
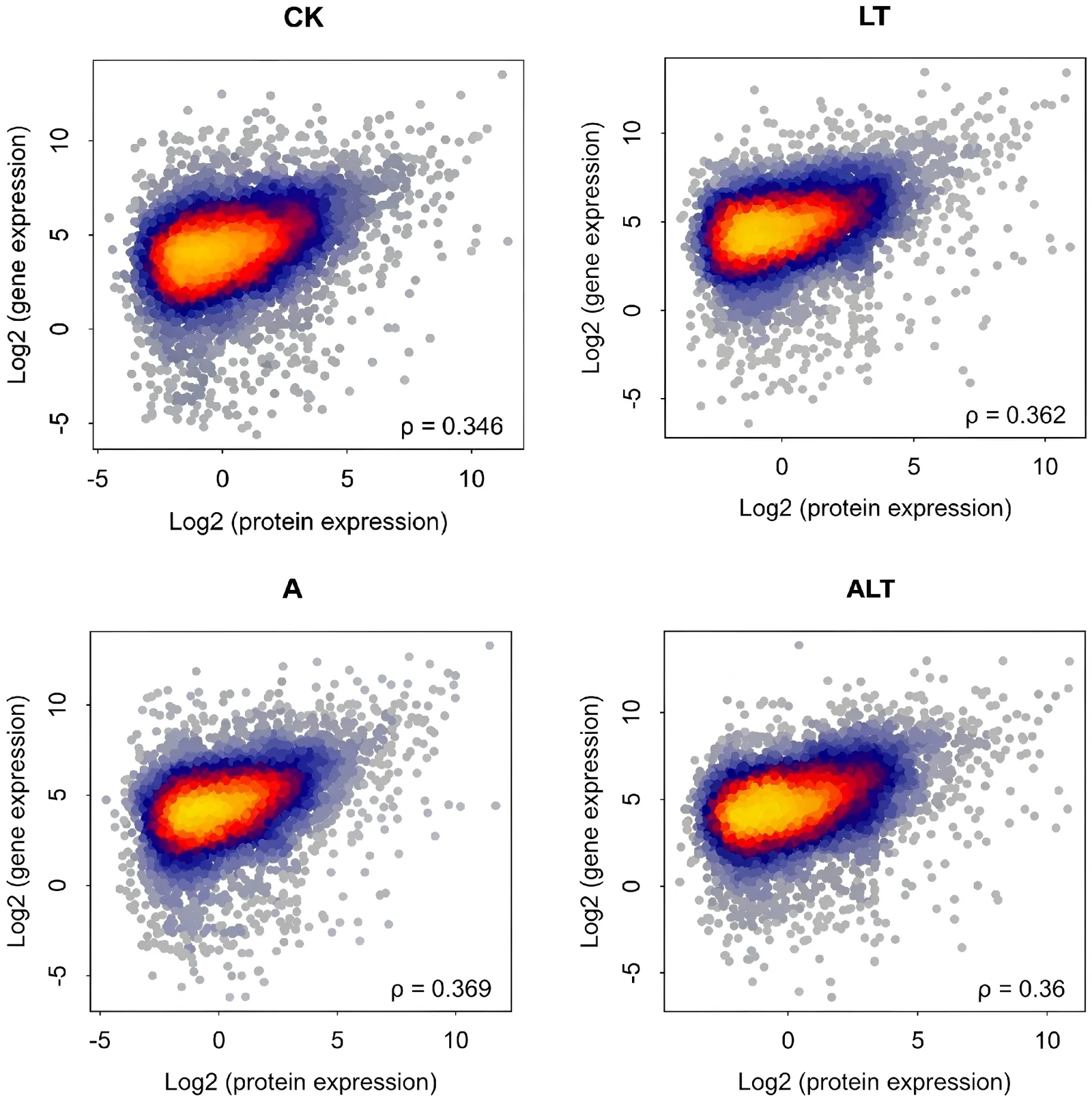
Correlation between transcriptome and proteome expression levels across four treatments in *R. yedoense* var. *poukhanense*. Density scatter plots show log_2_-transformed gene expression (y-axis) versus log_2_-transformed protein expression (x-axis) for shared genes detected in both omics layers. Spearman correlation coefficients (ρ) are indicated.

Gene-level expression correlation analysis across all 12 samples revealed a moderate but significant positive correlation between mRNA and protein expression levels (**Figure 9**). Among 5, 646 genes detected in both datasets, 1, 247 genes (22.1%) showed high concordance, including photosynthesis-related genes and core stress-responsive genes, suggesting tight transcriptional control of these essential functions. In contrast, 1, 876 genes (33.2%) showed discordant patterns, indicating substantial post-transcriptional regulation. Discordant genes were significantly enriched in “regulation of gene expression” and “protein phosphorylation”, suggesting that signaling and regulatory genes are preferentially subject to translational and post-translational control.

#### 3.6.2 Transcriptome-proteome discordance

Integrated transcriptome-proteome analysis across four pairwise comparisons revealed consistently weak transcript-protein correlations, indicative of pervasive post-transcriptional regulation (**Supplementary Figure 5**).

Pearson correlation coefficients between log_2_-transformed protein and gene fold changes were 0.016 for LT vs CK, 0.101 for A vs CK, 0.101 for ALT vs LT, and 0.104 for ALT vs A-all approaching zero and substantially lower than the theoretical expectation if changes were perfectly coupled. Nine-quadrant classification revealed that concordantly regulated genes (both transcript and protein significant in the same direction) comprised only a small fraction of the shared detected genes: 227 (4.1%) in LT vs CK (80 up, 147 down), 92 (1.7%) in A vs CK (46 up, 46 down), 56 (1.0%) in ALT vs LT (36 up, 20 down), and 225 (4.1%) in ALT vs A (96 up, 129 down). This limited concordance demonstrates that the majority of transcriptomic changes are not directly mirrored at the proteomic level, highlighting dominant roles of translational regulation, protein turnover, and time-lag effects in shaping the cold stress response. Notably, A vs CK and ALT vs LT exhibited the lowest concordance rates (~1.0-1.7%), suggesting that ABA-mediated responses involve particularly pronounced post-transcriptional buffering, whereas cold stress alone (LT vs CK) and the full combined treatment (ALT vs A) produced somewhat higher but still minor concordance (~4.1%), consistent with more direct stress-induced coupling between transcription and translation under severe physiological challenge.

#### 3.6.3 Protein-to-transcript ratio analysis

**Figure 10:**
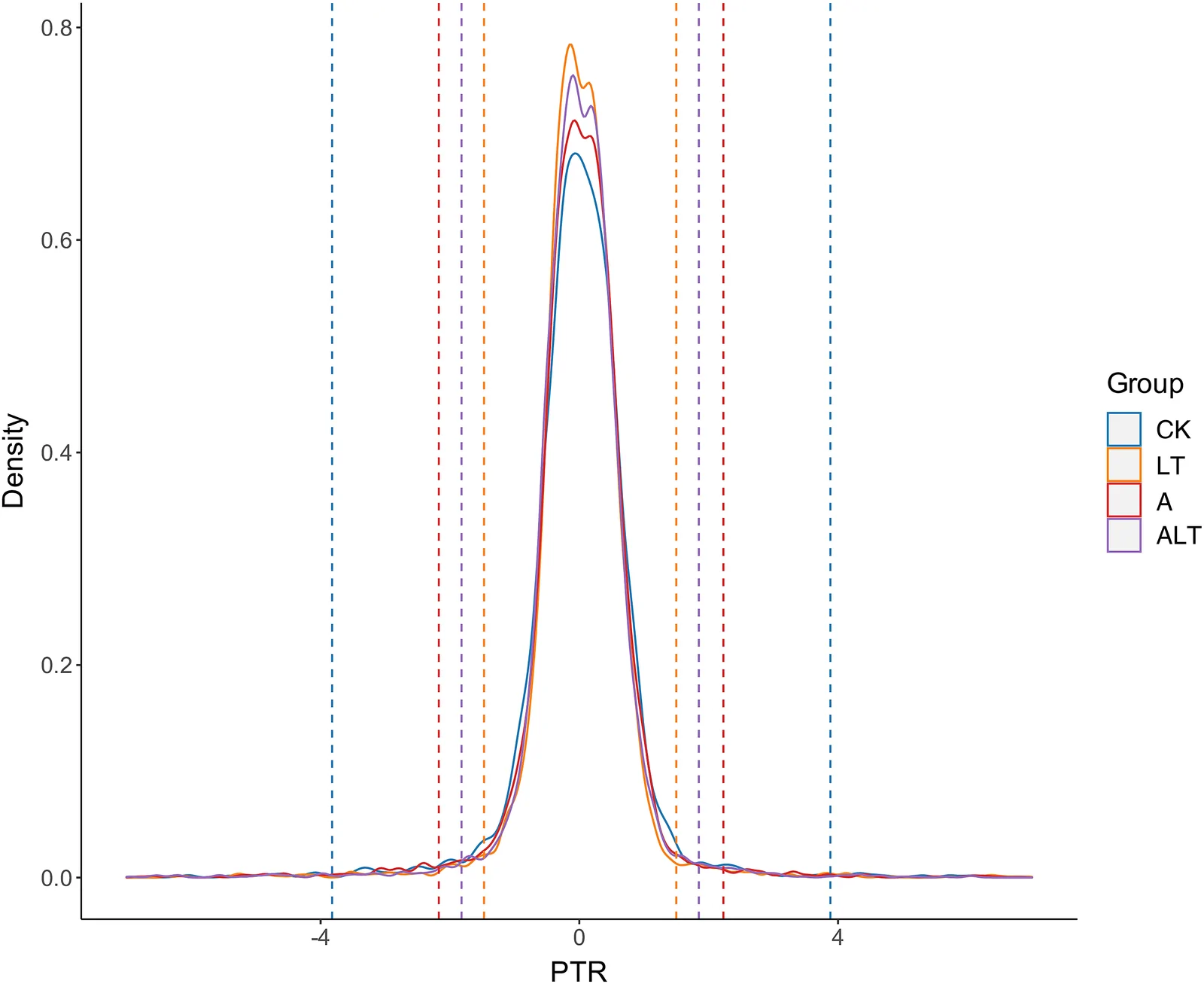
PTR density distribution across four treatments in *R. yedoense* var. *poukhanense*.

Protein-to-transcript ratio (PTR) analysis was performed to quantify translational efficiency remodeling across treatments, calculated as PTR = log_2_(protein fold change) - log_2_(transcript fold change) for 5, 645 genes detected in both omics layers (**Figure 10**). A total of 455 genes exhibited |PTR| > 1 in LT vs CK (221 high-PTR, 234 low-PTR), 594 genes in A vs CK (283 high-PTR, 311 low-PTR), and 505 genes in ALT vs CK (261 high-PTR, 244 low-PTR), demonstrating that translational efficiency remodeling is a pervasive feature of both cold and ABA responses. Notably, A vs CK exhibited the largest number of extreme-PTR genes (|PTR| > 2: 227), exceeding both LT vs CK (173) and ALT vs CK (176), indicating that ABA alone induces substantial translational reconfiguration even under non-stress conditions. High-PTR genes (PTR > 1) showed protein accumulation beyond transcriptional change; low-PTR genes (PTR < -1) showed protein decrease beyond transcriptional change.

### 3.7 qRT-PCR validation of transcriptome results

**Figure 11:**
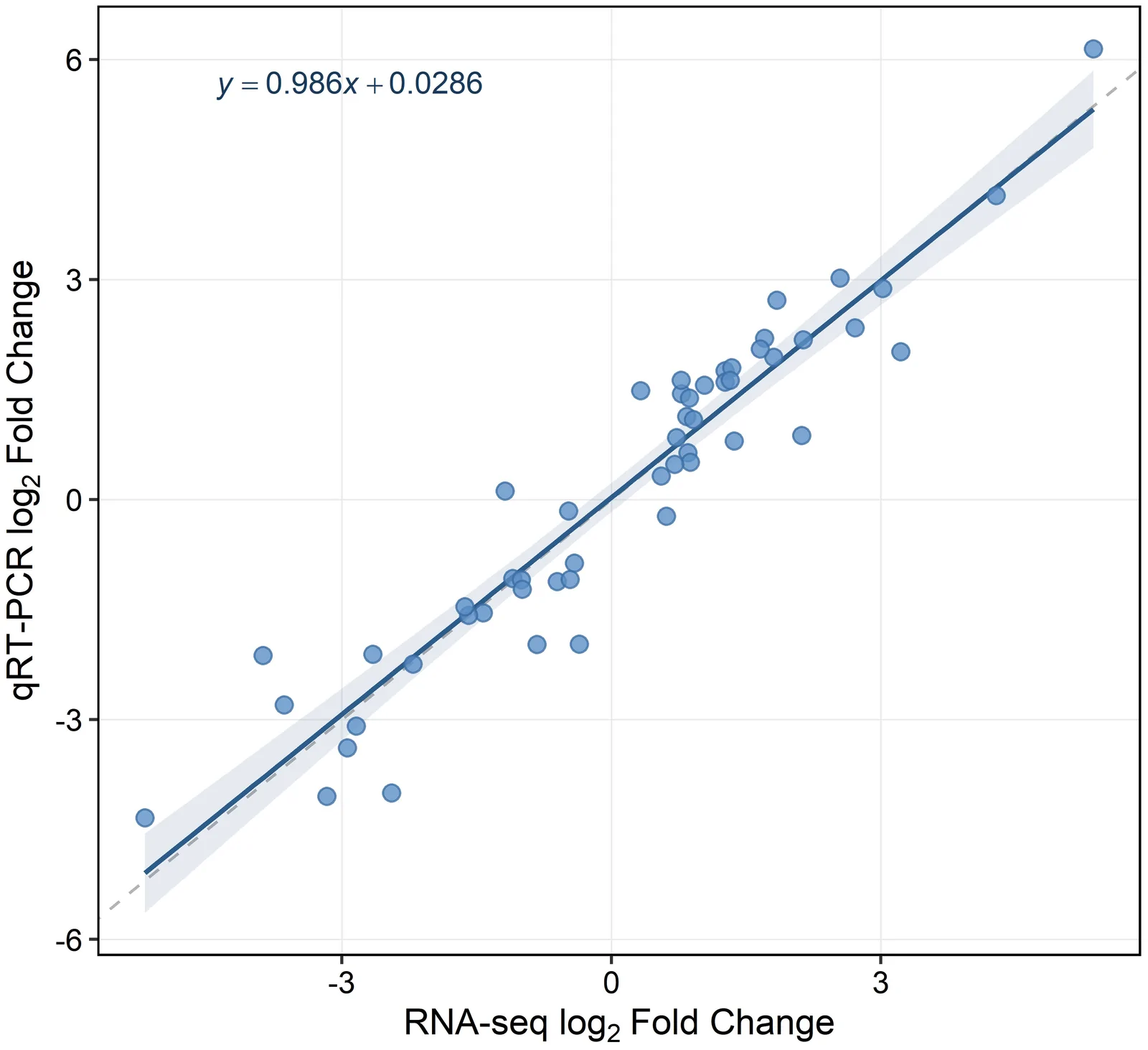
Validation of expression results using qRT-PCR representing the correlation between qRT-PCR and RNA-seq expression results for 15 selected DEGs from *R. yedoense* var. *poukhanense* under cold stress. Pearson R^2^ = 0.850, n = 15, *P* < 0.001.

To validate transcriptome data results, 15 DEGs involved in ABA signaling, antioxidant defense, cold response, and photosynthesis were selected for quantitative real-time PCR analysis (**Supplementary Table 2**). The qRT-PCR results showed a similar expression trend to the RNA-seq data. Pearson correlation analysis revealed a significant positive correlation between RNA-seq and qRT-PCR (r = 0.9219, R² = 0.8500, P < 0.001, n = 52), confirming the reliability of the transcriptome data (**Figure 11**; **Supplementary Figure 6**).

## 4 Discussion

### 4.1 ABA protects photosynthetic system under subzero temperature

Our data reveal that freezing at -4°C caused severe depletion of all four photosynthetic pigments (68-76% reduction), with Chl b showing greater sensitivity than Chl a (75.8% versus 68.2%). This hierarchy reflects the established lability of LHCII-associated pigments during thylakoid membrane disruption. The magnitude of loss at -4°C substantially exceeds that reported at 4°C (Huang et al., 2017), underscoring a non-linear severity-damage relationship not previously quantified in this species. Cold-induced pigment degradation involves multiple converging pathways: low temperatures impair chlorophyll biosynthesis by suppressing δ-aminolevulinic acid dehydratase and protochlorophyllide reductase activities while accelerating degradation via chlorophyllase activation and oxidative cleavage (Tanaka and Ito, 2025).

The partial preservation of pigments in ABA-pretreated seedlings (35-50% improvement over LT) demonstrates that exogenous ABA confers photoprotection under subzero conditions, which is similar to the research conducted on *C. japonica* (Fan et al., 2022). The protective mechanism likely involves ABA-mediated enhancement of non-photochemical quenching capacity through retention of xanthophyll pigment, which function in thermal dissipation of excess excitation energy and ROS scavenging (Huang et al., 2017). Notably, the accessory pigments showed the greatest relative improvement (50% each), suggesting that ABA preferentially protects components of the photoprotective apparatus. The incomplete recovery of chlorophylls in the ALT group indicates fundamental constraints on ABA-mediated protection at -4°C, where direct mechanical damage through ice formation and profound metabolic inhibition exceed the protective capacity of ABA signaling. In *citrus*, ABA similarly relieved chlorophyll degradation under cold stress but did not restore pigment contents to control levels (Mahmoud et al., 2024), suggesting that the quantitative limits of ABA-mediated photoprotection are conserved across diverse plant lineages. The phenotype data confirm that ABA shifts the threshold of visible damage without eliminating it.

### 4.2 ABA enhances osmotic regulation capacity

Our physiological data reveal a striking asymmetry in osmolyte responses to ABA priming: while SS accumulated to 349% above control, SP decreased by 29.8% in ALT relative to LT. This SS/SP divergence suggests a metabolic trade-off between energetically efficient sugar biosynthesis and nitrogen-intensive protein synthesis.

Low temperature was the dominant driver of SS accumulation, with ABA providing only modest additional enhancement, consistent with the temperature-dependence of starch hydrolysis and sucrose metabolism enzymes that operate independently of hormonal signaling. In *C. japonica*, coordinated upregulation of starch and sucrose metabolism pathways contributed substantially to cold tolerance divergence between varieties (Fan et al., 2022). The 157% increase in SS induced by ABA alone at normal temperature indicates that ABA can activate sugar accumulation through hormone-specific pathways, likely involving induction of sucrose phosphate synthase and trehalose-6-phosphate synthase expression (Shu et al., 2023). Under severe freezing, the near-saturation of SS accumulation in LT plants appears to constrain further ABA-mediated enhancement.

The substantial SP accumulation in both A and LT groups indicates that both ABA signaling and cold stress independently activate stress-related protein synthesis, including dehydrins, LEA proteins, and heat shock proteins that function as molecular chaperones and membrane stabilizers (Guo et al., 2024). This apparent protein catabolism may represent reallocation of nitrogen and carbon resources toward energetically more efficient osmolytes and ATP generation through amino acid oxidation (Hildebrandt, 2018).

The massive proline accumulation under freezing stress and its near-saturation in the ALT group indicates that proline biosynthetic capacity at -4°C is saturated by cold-induced P5CS activation, leaving limited scope for further ABA-mediated enhancement. The 85% increase in proline induced by ABA alone at normal temperature is consistent with ABA-mediated upregulation of P5CS, the rate-limiting enzyme in proline biosynthesis (Savouré et al., 1997). These differential responses demonstrate that ABA modulates distinct metabolic branches of the osmotic adjustment network in a context-dependent manner.

The contrasting effects on soluble protein versus soluble sugar are particularly informative. While both osmolytes serve cryoprotective functions, their biosynthesis competes for carbon skeletons and energy resources. Under severe freezing, the shift from protein accumulation in LT to relative protein decrease in ALT, accompanied by continued sugar accumulation, may represent a metabolic trade-off favoring rapid, energetically efficient osmolyte production over protein synthesis, which requires greater ATP and nitrogen investment. Such metabolic reprogramming, though reducing SP content, may optimize overall osmoprotective efficiency under energetically constrained conditions.

### 4.3 ABA activates antioxidant defense system

The differential responses of the three antioxidant enzymes to ABA and cold stress reveal a hierarchical organization of the antioxidant defense system. Under normal temperature, ABA induced all three enzymes but with markedly different magnitudes: CAT> SOD > POD, establishing a primed defense state. This hierarchy was maintained under freezing stress, where ABA-mediated recovery followed the same order. The preferential responsiveness of CAT identifies this enzyme as the primary ABA-regulated component of the antioxidant system, consistent with findings in *R. chrysanthum* where CAT and SOD are central to cold defense (Zhang et al., 2023).

The preferential recovery of CAT activity may reflect ABA-specific signaling to peroxisomal antioxidant pathways. CAT is localized exclusively to peroxisomes, where it catalyzes H_2_O_2_ dismutation without consuming reducing equivalents, making it energetically efficient under conditions where NADPH availability is limiting (Mittler, 2002). The observation that CAT showed both the greatest cold-induced inhibition and the strongest ABA recovery suggests that this enzyme is simultaneously the most vulnerable to freezing damage and the most responsive to ABA-mediated protective signaling.

The 28.7% reduction in MDA content in ABA-pretreated seedlings under freezing stress provides direct biochemical evidence that ABA alleviates membrane lipid peroxidation. The reduction of MDA in the ALT group is attributable to partially recovered antioxidant enzyme activities, particularly CAT, which reduced H_2_O_2_ accumulation and limited hydroxyl radical-mediated lipid peroxidation chain reactions. The persistence of elevated MDA in the ALT group underscores the quantitative limits of ABA-mediated protection at -4°C, where multiple mechanisms of cellular injury operate concurrently and cannot be fully mitigated by hormonal pretreatment alone (Li et al., 2025).

The incomplete recovery of antioxidant enzyme activities in the ALT group-with all three enzymes remaining 27-47% below baseline-highlights fundamental constraints on ABA-mediated freezing tolerance enhancement. Severe freezing impairs protein synthesis machinery, including ribosomal function and translational initiation, constraining *de novo* enzyme synthesis even when ABA signaling is activated (Wang et al., 2017). Freezing-induced membrane disruption can also impair subcellular compartmentalization required for antioxidant enzyme function, and the energetic cost of maintaining high enzyme activities may exceed the ATP-generating capacity of mitochondria under freezing conditions (Kerbler et al., 2019). Prior *Rhododendron* studies at 4°C reported substantial maintenance or even enhancement of antioxidant enzyme activities under cold stress (Zhang et al., 2023), indicating that the antioxidant system operates effectively above freezing but becomes progressively impaired at subzero temperatures. Future studies should explore temperature-dose interaction effects and time-course dynamics to optimize ABA application protocols for practical cold protection.

### 4.4 Post-transcriptional regulation and protein-level response

Integration of transcriptomic and proteomic datasets revealed remarkably low mRNA-protein concordance. This pervasive disconnect reflects translational regulation, protein turnover via the ubiquitin-proteasome system and autophagy, and post-translational modifications not captured in the present workflow (Keller et al., 2018). Extensive phosphorylation changes in *R. chrysanthum* cold-responsive proteins, emphasizing that PTM-driven regulation constitutes a major control layer beyond abundance-based detection (Liu et al., 2022).

The proteomic data revealed a distinct quantitative profile. ALT vs CK yielded 1, 461 DEPs-the highest of all comparisons-while LT vs CK produced 1, 244 DEPs with a pronounced downregulation bias (468 upregulated, 776 downregulated), contrasting with the transcriptome where upregulation dominated. This suggests translational inhibition or enhanced protein degradation as a major regulatory node at -4°C, potentially reflecting an energy-conservation strategy (Wang et al., 2017). The ABA-mediated recovery of CAT activity (+44%) further indicates that ABA selectively stabilizes specific defense proteins while allowing broader proteomic repression. Zhang reported co-regulation of ABA, MAPK, and Ca^2+^ signaling at the protein level in *R. chrysanthum*, reinforcing that ABA modulates protein abundance through multiple mechanisms (Zhang et al., 2023).

High-PTR genes represent proteins that accumulate beyond their transcriptional change, reflecting enhanced translation or increased protein stability, whereas low-PTR genes indicate translational repression or accelerated protein degradation. The comparable numbers of extreme-PTR genes in LT and ALT suggest that ABA pretreatment does not globally suppress cold-induced translational remodeling but rather redirects it toward specific functional modules, consistent with the priming model observed at the transcriptional and proteomic levels.

The limited transcript–protein concordance observed in this study (1.0-4.1% across pairwise comparisons) primarily reflects genuine biological processes, including translational regulation, protein turnover, and post-translational modifications, which are well-documented contributors to transcript–protein discordance in plant stress responses. Nevertheless, we acknowledge that technical factors associated with cross-species alignment may introduce a modest additional contribution to the observed discordance. Specifically, mis-assignment of reads to paralogous loci or incomplete gene models in the congeneric *R. simsii* reference could generate annotation-driven noise that inflates the discordant and unchanged categories in the nine-quadrant analysis. We emphasize that the dominant driver of the low concordance is biological post-transcriptional regulation, as supported by the high qRT-PCR validation rate (R^2^ = 0.8500) and the coherent physiological-proteome-transcriptome patterns observed across all treatment groups. Full disentanglement of biological and technical contributions will ultimately benefit from a species-specific reference genome for *R. yedoense* var. *poukhanense*.

### 4.5 ABA-mediated cold tolerance network

The multi-omics and physiological data support a conceptual model of ABA-mediated cold tolerance across four hierarchical layers: signal perception (membrane rigidification, Ca^2+^ influx, ROS generation), transcriptional reprogramming (>8, 000 DEGs encompassing hormone signaling, MAPK cascades, and secondary metabolism, attenuated by ABA pre-treatment), post-transcriptional regulation (translational control, selective mRNA degradation, and protein turnover decoupling transcript from protein abundance), and physiological execution (partial pigment protection, 349% soluble sugar accumulation, enhanced CAT activity, and 29% MDA reduction).

This architecture explains why ABA confers partial rather than complete protection at -4°C: persistent MDA accumulation (still 2× control) indicates that subzero severity exceeds exogenous ABA buffering capacity. Gusta summarized four mechanisms of ABA-enhanced cold tolerance-osmotic regulation, antioxidant activation, membrane stabilization, and ABF-mediated gene expression-with the present data supporting the first three (Gusta et al., 2005). The CBF pathway, positioned as a hormone cross-talk node, integrates multiple inputs beyond ABA alone (Barrero-Gil and Salinas, 2017).

A critical constraint is reliance on reference databases biased toward model species. The use of a congeneric reference genome (*R. simsii*, PRJNA588298) rather than the native genome of *R. yedoense* var. *poukhanense* introduced limitations in annotation completeness. Species-specific transcripts, lineage-specific regulatory elements, and fine-tuned gene models may not be fully captured by cross-species mapping, which partially explains why some canonical ABA signaling components (e.g., PYL, SnRK2, ABF) and ICE1-CBF-COR cascade members were not confidently identified—a challenge shared with other non-model species studies (Shao et al., 2024). A well-annotated reference genome would substantially advance functional interpretation; until then, integrated multi-omics approaches offer the most robust path forward.

### 4.6 Limitations of cross-species transcriptome and proteome analysis

It is important to acknowledge that the transcriptome and proteome analyses in this study were performed using the *R. simsii* reference genome (Yang et al., 2020) because a chromosome-level assembly for *R. yedoense* var. *poukhanense* is not currently available. While *R. simsii* and *R. yedoense* var. *poukhanense* belong to the same genus and share a relatively recent common ancestor, cross-species alignment introduces several systematic limitations that warrant cautious interpretation of the results.

First, species-specific transcripts—genes uniquely present or substantially diverged in *R. yedoense* var. *poukhanense*—may be entirely lost or underrepresented in the alignment. HISAT2 mapping statistics revealed an overall mapping rate of 77.60% and a uniquely mapped rate of 73.78% across all 12 samples, indicating that approximately 22% of clean reads could not be confidently assigned to the *R. simsii* reference. These unmapped reads likely contain sequences from *R. yedoense* var. *poukhanense*-specific genes or highly diverged orthologs that are absent or too divergent in the R. simsii genome. Consequently, the DEGs and DEPs reported in this study likely represent the subset of cold-responsive genes that are conserved between *R. simsii* and *R. yedoense* var. *poukhanense*, and additional species-specific cold-adaptive mechanisms may await discovery with a dedicated reference genome.

Second, annotation bias arising from the *R. simsii* gene models may affect functional interpretation. The reference database (pep.fa) contains 32, 999 protein-coding sequences annotated from the *R. simsii* genome (NCBI BioProject PRJNA588298), and KEGG/GO enrichment analyses relied on these annotations. However, gene annotation quality is known to vary substantially even among well-assembled plant genomes, and structural annotation errors—including missed exons, incorrect splice variants, or fused gene models—can propagate into downstream functional analyses (Hirsch et al., 2015). In non-model species where experimental validation of gene models is scarce, such annotation-dependent biases are difficult to quantify and correct.

Third, the inability to distinguish paralogous genes represents a particularly significant limitation in *Rhododendron*. The Ericaceae family has undergone at least two ancient whole-genome duplication (WGD) events (Soza et al., 2019), resulting in extensive retention of paralogous gene copies. When RNA-seq reads from *R. yedoense* var. *poukhanense* are aligned to the *R. simsii* reference, reads originating from recently duplicated paralogs with high sequence similarity may map to a single reference locus, effectively collapsing expression signals from multiple gene copies into one. Hirsch et al. (2015) demonstrated that in plant genomes with recent WGD, over 25% of genes can exhibit >20% deviation from expected expression levels due to paralog mapping ambiguity. This limitation is especially relevant for gene families central to cold stress response—such as dehydrins, LEA proteins, and antioxidant enzymes—which are frequently expanded through tandem duplication and may have functionally diverged between *R. simsii* and *R. yedoense* var. *poukhanense*.

Despite these limitations, several factors support the robustness of the core findings. The overall mapping rate of 77.60% is consistent with cross-species RNA-seq studies in congeneric plant species, and the high concordance between transcriptome and qRT-PCR data (R^2^ = 0.8500) validates the quantitative reliability of the aligned subset. Furthermore, the integrated multi-omics approach—combining physiology, transcriptome, and proteome data—provides cross-validating evidence that reduces dependence on any single data layer. Nevertheless, the generation of a chromosome-level reference genome for *R. yedoense* var. *poukhanense* remains a critical priority for future research, as it would enable detection of species-specific genes, improve annotation accuracy, and resolve paralog-specific expression patterns that are currently inaccessible.

## 5 Conclusion

This study demonstrates that exogenous ABA (30 mg·L^-1^) enhances freezing tolerance in *R. yedoense* var. *poukhanense* at -4°C through multi-layered molecular regulation, as evidenced by integrated physiological, transcriptomic, and proteomic analyses. Physiologically, ABA pretreatment reduced cold injury severity by one grade, partially preserved photosynthetic pigments, promoted osmolyte accumulation, and activated antioxidant defenses while reducing lipid peroxidation by 28.7%. Transcriptome analysis revealed massive cold-induced reprogramming that was attenuated by ABA pretreatment, with the overall DEG number reduced by 23% in ALT vs CK relative to LT vs CK. The CBF cold regulon and ABA signaling core pathways were selectively preserved while ROS-activated WRKY/NAC modules were attenuated, consistent with an ABA priming effect established during pretreatment. Proteome analysis identified 1, 244 DEPs in LT vs CK, revealing dominant ribosomal suppression and translational inhibition under freezing, which was dramatically restored by ABA pretreatment. Joint transcriptome-proteome analysis revealed pervasive post-transcriptional regulation, with only 1.0-4.1% transcript-protein concordance across pairwise comparisons, complete KEGG pathway decoupling, and extensive translational efficiency remodeling evidenced by PTR analysis. These findings establish ABA-mediated cold priming as a hierarchical process integrating transcriptional buffering, translational reprogramming, and functional reallocation from photosynthesis to stress protection, providing the multi-omics framework for ABA-enhanced freezing tolerance in evergreen ornamental shrubs and theoretical support for ABA-based winter protection strategies.

## Data Availability

The original contributions presented in the study are publicly available. The transcriptome sequencing data reported in this paper have been submitted to the NCBI Sequence Read Archive (SRA) (BioProject accession number: PRJNA1483052). The mass spectrometry proteomics data have been deposited to the ProteomeXchange Consortium via the iProX partner repository with the dataset identifier PXD081457.
