# Supplementary material for: Exogenous ABA enhances cold tolerance of Rhododendron yedoense var. poukhanense under subzero temperature: integrating physiology, transcriptome, and proteome
Source: Front Plant Sci. 2026 Aug 28;17:1921248. doi: 10.3389/fpls.2026.1921248 (PMC13561794; doi:10.3389/fpls.2026.1921248)
Supplement: Supplementary file 1 [file DataSheet1.pdf]

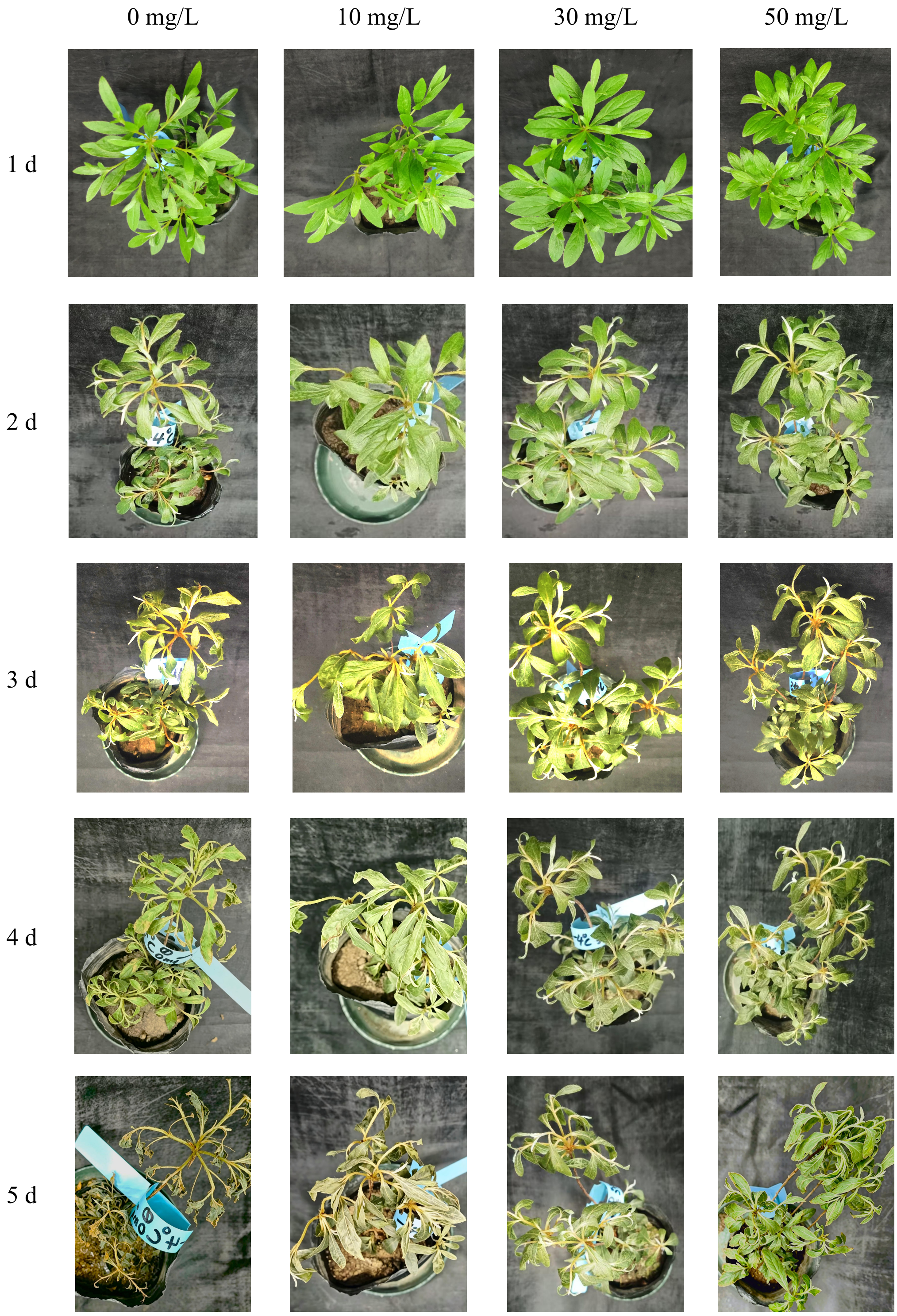

Supplementary Figure 1 ABA concentration screening pre-experiment. Phenotypic responses of R. yedoense var. poukhanense to 0, 10, 30, and 50 mg·L^{-1} ABA under -4°C freezing stress over 5 days. The 30 mg·L^{-1} treatment (highlighted) showed the least wilting and chlorosis by Day 5 and was selected as the optimal concentration for subsequent experiments.
