# Supplementary material for: Exogenous ABA enhances cold tolerance of Rhododendron yedoense var. poukhanense under subzero temperature: integrating physiology, transcriptome, and proteome
Source: Front Plant Sci. 2026 Aug 28;17:1921248. doi: 10.3389/fpls.2026.1921248 (PMC13561794; doi:10.3389/fpls.2026.1921248)
Supplement: Supplementary file 2 [file DataSheet2.pdf]

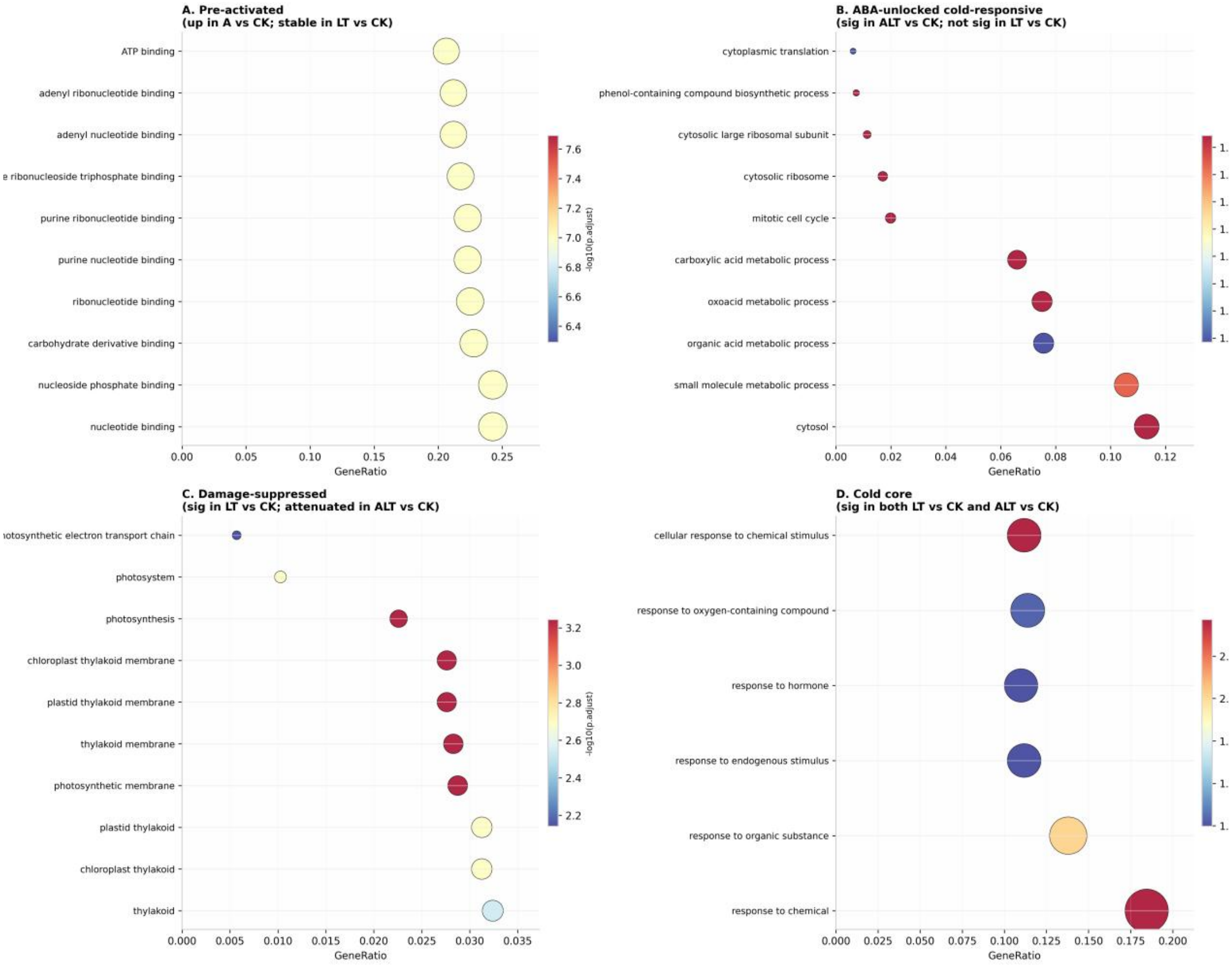

Supplementary Figure 2 GO enrichment bubble plots of ABA priming-associated gene subsets. (A) Pre-activated subset (1,397 genes upregulated in A vs CK but unchanged in LT vs CK). (B) ABA-unlocked cold-responsive subset (2,224 genes significant in ALT vs CK but not in LT vs CK). (C) Damage-suppressed subset (5,619 genes significant in LT vs CK but attenuated in ALT vs CK). (D) Cold core subset (4,114 genes significant in both LT vs CK and ALT vs CK with same direction). The ABA-specific subset (483 genes) did not yield significant GO enrichment and is omitted. Bubble size represents the number of genes enriched in each GO term (Gene Count); color represents -$\log_{10}$(adjusted p-value). GO enrichment was performed using Fisher's exact test with BH correction (FDR < 0.05).
