# Supplementary material for: Exogenous ABA enhances cold tolerance of Rhododendron yedoense var. poukhanense under subzero temperature: integrating physiology, transcriptome, and proteome
Source: Front Plant Sci. 2026 Aug 28;17:1921248. doi: 10.3389/fpls.2026.1921248 (PMC13561794; doi:10.3389/fpls.2026.1921248)
Supplement: Supplementary file 3 [file DataSheet3.pdf]

(A)

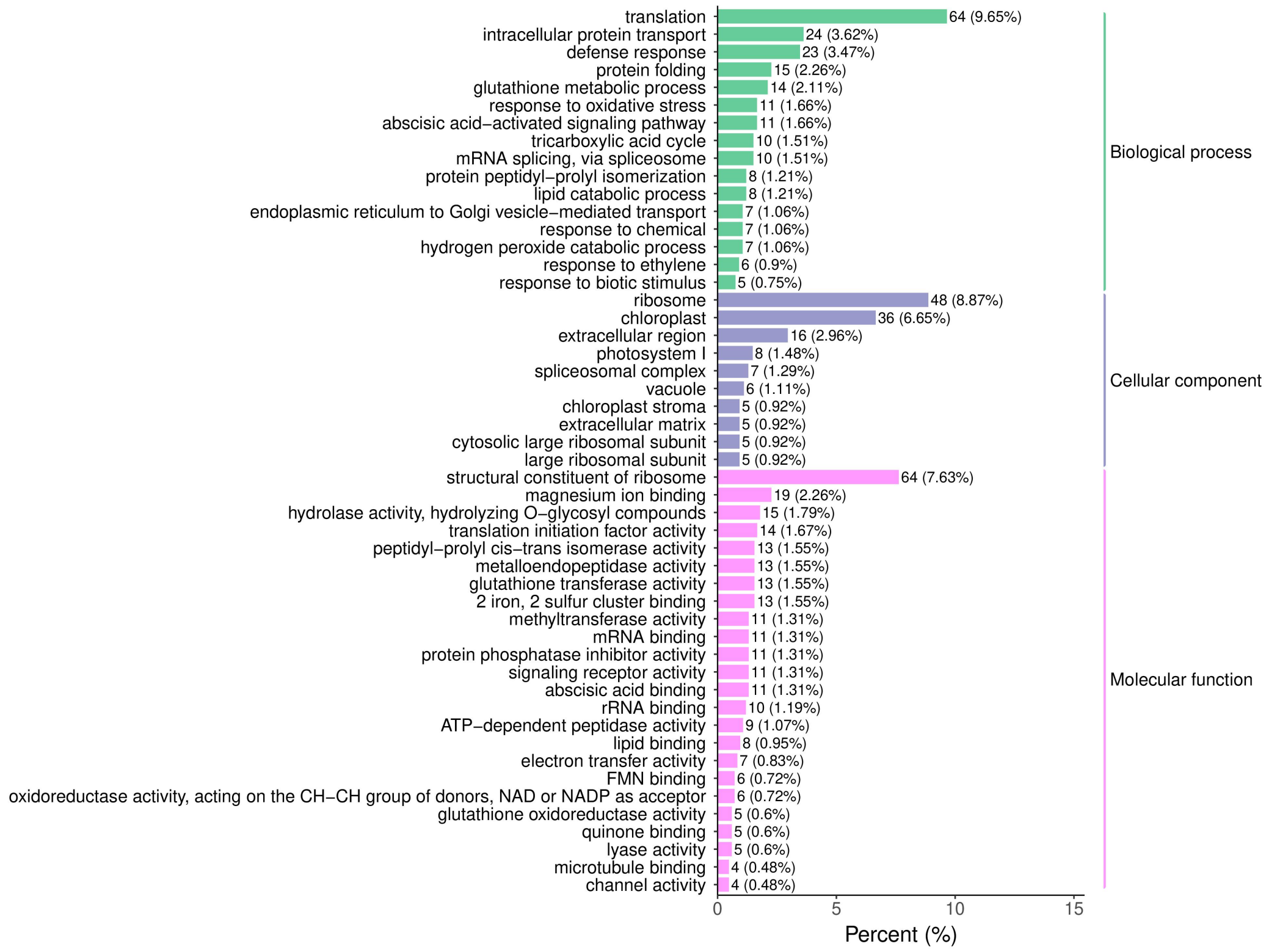
translation 64 (9.65%)
intracellular protein transport 24 (3.62%)
defense response 23 (3.47%)
protein folding 15 (2.26%)
glutathione metabolic process 14 (2.11%)
response to oxidative stress 11 (1.66%)
abscisic acid−activated signaling pathway 11 (1.66%)
tricarboxylic acid cycle 10 (1.51%)
mRNA splicing, via spliceosome 10 (1.51%)
protein peptidyl−prolyl isomerization 8 (1.21%)
lipid catabolic process 8 (1.21%)
endoplasmic reticulum to Golgi vesicle−mediated transport 7 (1.06%)
response to chemical 7 (1.06%)
hydrogen peroxide catabolic process 7 (1.06%)
response to ethylene 6 (0.9%)
response to biotic stimulus 5 (0.75%)
ribosome 48 (8.87%)
chloroplast 36 (6.65%)
extracellular region 16 (2.96%)
photosystem I 8 (1.48%)
spliceosomal complex 7 (1.29%)
vacuole 6 (1.11%)
chloroplast stroma 5 (0.92%)
extracellular matrix 5 (0.92%)
cytosolic large ribosomal subunit 5 (0.92%)
large ribosomal subunit 5 (0.92%)
structural constituent of ribosome 64 (7.63%)
magnesium ion binding 19 (2.26%)
hydrolase activity, hydrolyzing O−glycosyl compounds 15 (1.79%)
translation initiation factor activity 14 (1.67%)
peptidyl−prolyl cis−trans isomerase activity 13 (1.55%)
metalloendopeptidase activity 13 (1.55%)
glutathione transferase activity 13 (1.55%)
2 iron, 2 sulfur cluster binding 13 (1.55%)
methyltransferase activity 11 (1.31%)
mRNA binding 11 (1.31%)
protein phosphatase inhibitor activity 11 (1.31%)
signaling receptor activity 11 (1.31%)
abscisic acid binding 11 (1.31%)
rRNA binding 10 (1.19%)
ATP−dependent peptidase activity 9 (1.07%)
lipid binding 8 (0.95%)
electron transfer activity 7 (0.83%)
FMN binding 6 (0.72%)
oxidoreductase activity, acting on the CH−CH group of donors, NAD or NADP as acceptor 6 (0.72%)
glutathione oxidoreductase activity 5 (0.6%)
quinone binding 5 (0.6%)
lyase activity 5 (0.6%)
microtubule binding 4 (0.48%)
channel activity 4 (0.48%)
0
5
10
15
Percent (%)
Biological process
Cellular component
Molecular function

(B)

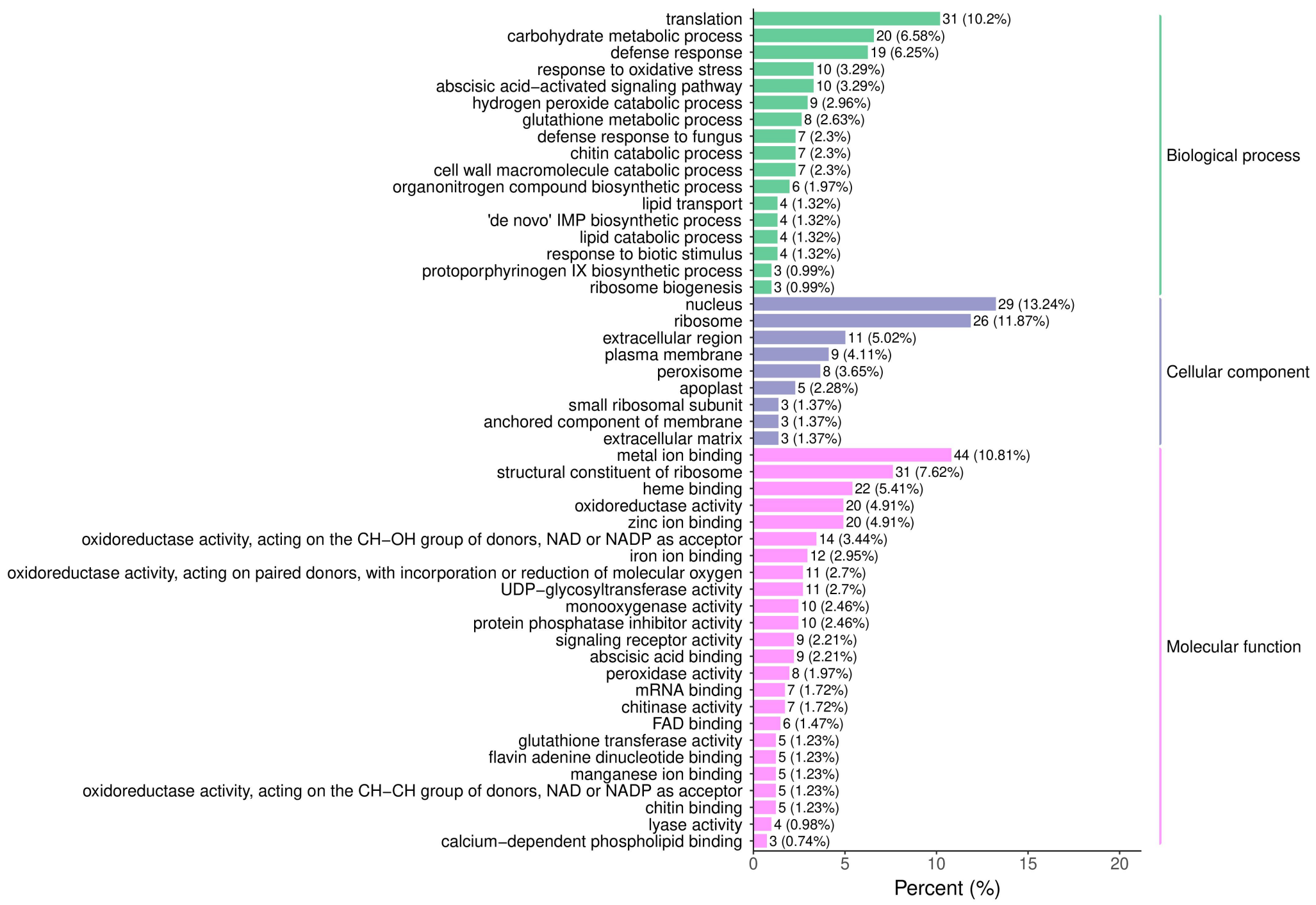
translation 31 (10.2%)
carbohydrate metabolic process 20 (6.58%)
defense response 19 (6.25%)
response to oxidative stress 10 (3.29%)
abscisic acid−activated signaling pathway 10 (3.29%)
hydrogen peroxide catabolic process 9 (2.96%)
glutathione metabolic process 8 (2.63%)
defense response to fungus 7 (2.3%)
chitin catabolic process 7 (2.3%)
cell wall macromolecule catabolic process 7 (2.3%)
organonitrogen compound biosynthetic process 6 (1.97%)
lipid transport 4 (1.32%)
'de novo' IMP biosynthetic process 4 (1.32%)
lipid catabolic process 4 (1.32%)
response to biotic stimulus 4 (1.32%)
protoporphyrinogen IX biosynthetic process 3 (0.99%)
ribosome biogenesis 3 (0.99%)
nucleus 29 (13.24%)
ribosome 26 (11.87%)
extracellular region 11 (5.02%)
plasma membrane 9 (4.11%)
peroxisome 8 (3.65%)
apoplast 5 (2.28%)
small ribosomal subunit 3 (1.37%)
anchored component of membrane 3 (1.37%)
extracellular matrix 3 (1.37%)
metal ion binding 44 (10.81%)
structural constituent of ribosome 31 (7.62%)
heme binding 22 (5.41%)
oxidoreductase activity 20 (4.91%)
zinc ion binding 20 (4.91%)
oxidoreductase activity, acting on the CH−OH group of donors, NAD or NADP as acceptor 14 (3.44%)
iron ion binding 12 (2.95%)
oxidoreductase activity, acting on paired donors, with incorporation or reduction of molecular oxygen 11 (2.7%)
UDP−glycosyltransferase activity 11 (2.7%)
monooxygenase activity 10 (2.46%)
protein phosphatase inhibitor activity 10 (2.46%)
signaling receptor activity 9 (2.21%)
abscisic acid binding 9 (2.21%)
peroxidase activity 8 (1.97%)
mRNA binding 7 (1.72%)
chitinase activity 7 (1.72%)
FAD binding 6 (1.47%)
glutathione transferase activity 5 (1.23%)
flavin adenine dinucleotide binding 5 (1.23%)
manganese ion binding 5 (1.23%)
oxidoreductase activity, acting on the CH−CH group of donors, NAD or NADP as acceptor 5 (1.23%)
chitin binding 5 (1.23%)
lyase activity 4 (0.98%)
calcium−dependent phospholipid binding 3 (0.74%)
0
5
10
15
20
Percent (%)
Biological process
Cellular component
Molecular function

(C)

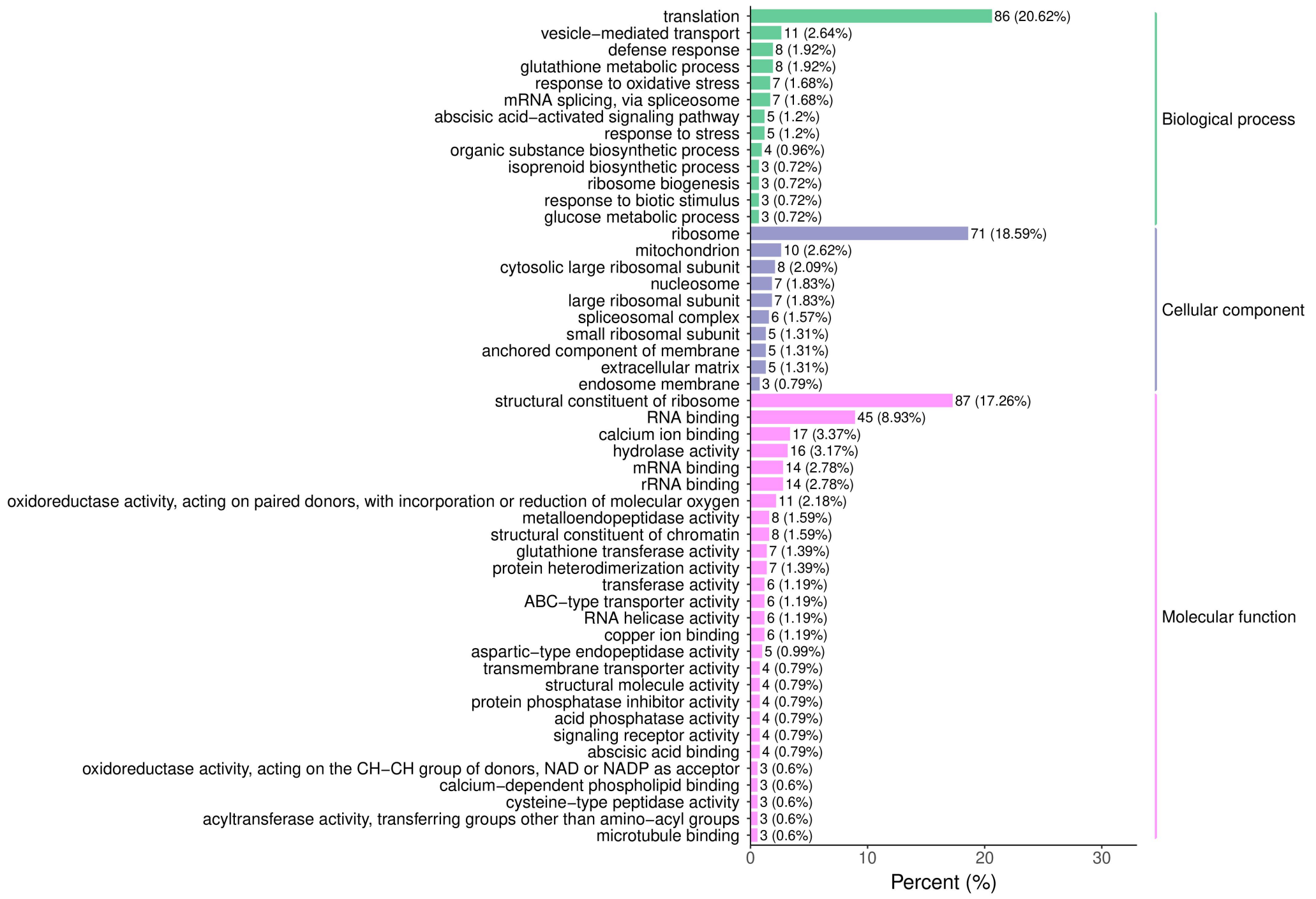

(D)

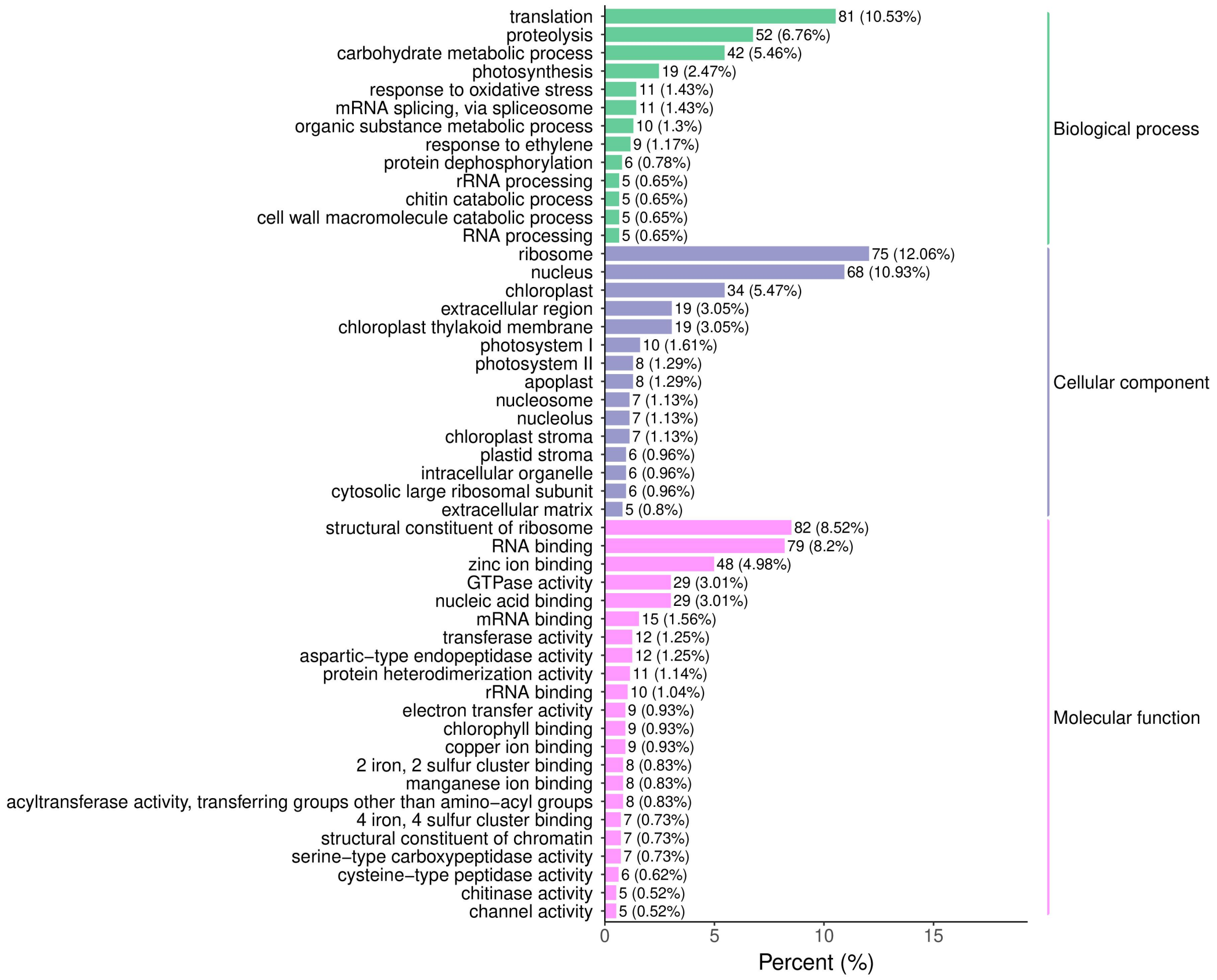

Supplementary Figure 3 GO enrichment analysis of DEPs across four pairwise comparisons in *R. yedoense* var. *poukhanense*. GO terms are categorized into Biological Process (BP), Cellular Component (CC), and Molecular Function (MF).

Bar length indicates the percentage of enriched DEPs within each GO category; numbers in parentheses show the absolute DEP count. (A) LT vs CK; (B) A vs CK; (C) ALT vs LT; (D) ALT vs A.
