# Supplementary material for: Exogenous ABA enhances cold tolerance of Rhododendron yedoense var. poukhanense under subzero temperature: integrating physiology, transcriptome, and proteome
Source: Front Plant Sci. 2026 Aug 28;17:1921248. doi: 10.3389/fpls.2026.1921248 (PMC13561794; doi:10.3389/fpls.2026.1921248)
Supplement: Supplementary file 4 [file DataSheet4.pdf]

(A)

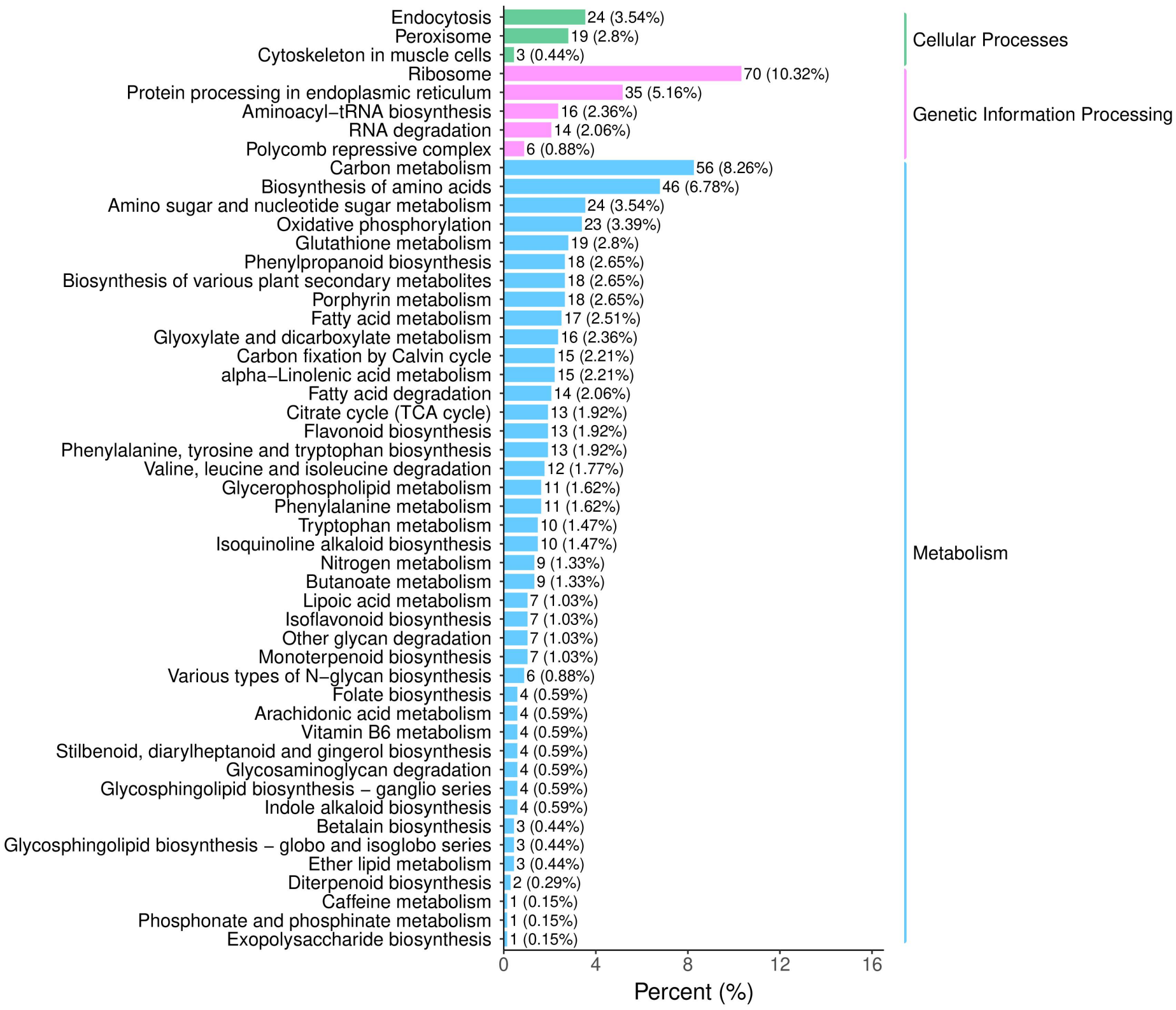
Endocytosis
24 (3.54%)
Peroxisome
19 (2.8%)
Cytoskeleton in muscle cells
3 (0.44%)
Ribosome
70 (10.32%)
Protein processing in endoplasmic reticulum
35 (5.16%)
Aminoacyl−tRNA biosynthesis
16 (2.36%)
RNA degradation
14 (2.06%)
Polycomb repressive complex
6 (0.88%)
Carbon metabolism
56 (8.26%)
Biosynthesis of amino acids
46 (6.78%)
Amino sugar and nucleotide sugar metabolism
24 (3.54%)
Oxidative phosphorylation
23 (3.39%)
Glutathione metabolism
19 (2.8%)
Phenylpropanoid biosynthesis
18 (2.65%)
Biosynthesis of various plant secondary metabolites
18 (2.65%)
Porphyrin metabolism
18 (2.65%)
Fatty acid metabolism
17 (2.51%)
Glyoxylate and dicarboxylate metabolism
16 (2.36%)
Carbon fixation by Calvin cycle
15 (2.21%)
alpha−Linolenic acid metabolism
15 (2.21%)
Fatty acid degradation
14 (2.06%)
Citrate cycle (TCA cycle)
13 (1.92%)
Flavonoid biosynthesis
13 (1.92%)
Phenylalanine, tyrosine and tryptophan biosynthesis
13 (1.92%)
Valine, leucine and isoleucine degradation
12 (1.77%)
Glycerophospholipid metabolism
11 (1.62%)
Phenylalanine metabolism
11 (1.62%)
Tryptophan metabolism
10 (1.47%)
Isoquinoline alkaloid biosynthesis
10 (1.47%)
Nitrogen metabolism
9 (1.33%)
Butanoate metabolism
9 (1.33%)
Lipoic acid metabolism
7 (1.03%)
Isoflavonoid biosynthesis
7 (1.03%)
Other glycan degradation
7 (1.03%)
Monoterpenoid biosynthesis
7 (1.03%)
Various types of N−glycan biosynthesis
6 (0.88%)
Folate biosynthesis
4 (0.59%)
Arachidonic acid metabolism
4 (0.59%)
Vitamin B6 metabolism
4 (0.59%)
Stilbenoid, diarylheptanoid and gingerol biosynthesis
4 (0.59%)
Glycosaminoglycan degradation
4 (0.59%)
Glycosphingolipid biosynthesis − ganglio series
4 (0.59%)
Indole alkaloid biosynthesis
4 (0.59%)
Betalain biosynthesis
3 (0.44%)
Glycosphingolipid biosynthesis − globo and isoglobo series
3 (0.44%)
Ether lipid metabolism
3 (0.44%)
Diterpenoid biosynthesis
2 (0.29%)
Caffeine metabolism
1 (0.15%)
Phosphonate and phosphinate metabolism
1 (0.15%)
Exopolysaccharide biosynthesis
1 (0.15%)
0
4
8
12
16
Percent (%)
Cellular Processes
Genetic Information Processing
Metabolism

(B)

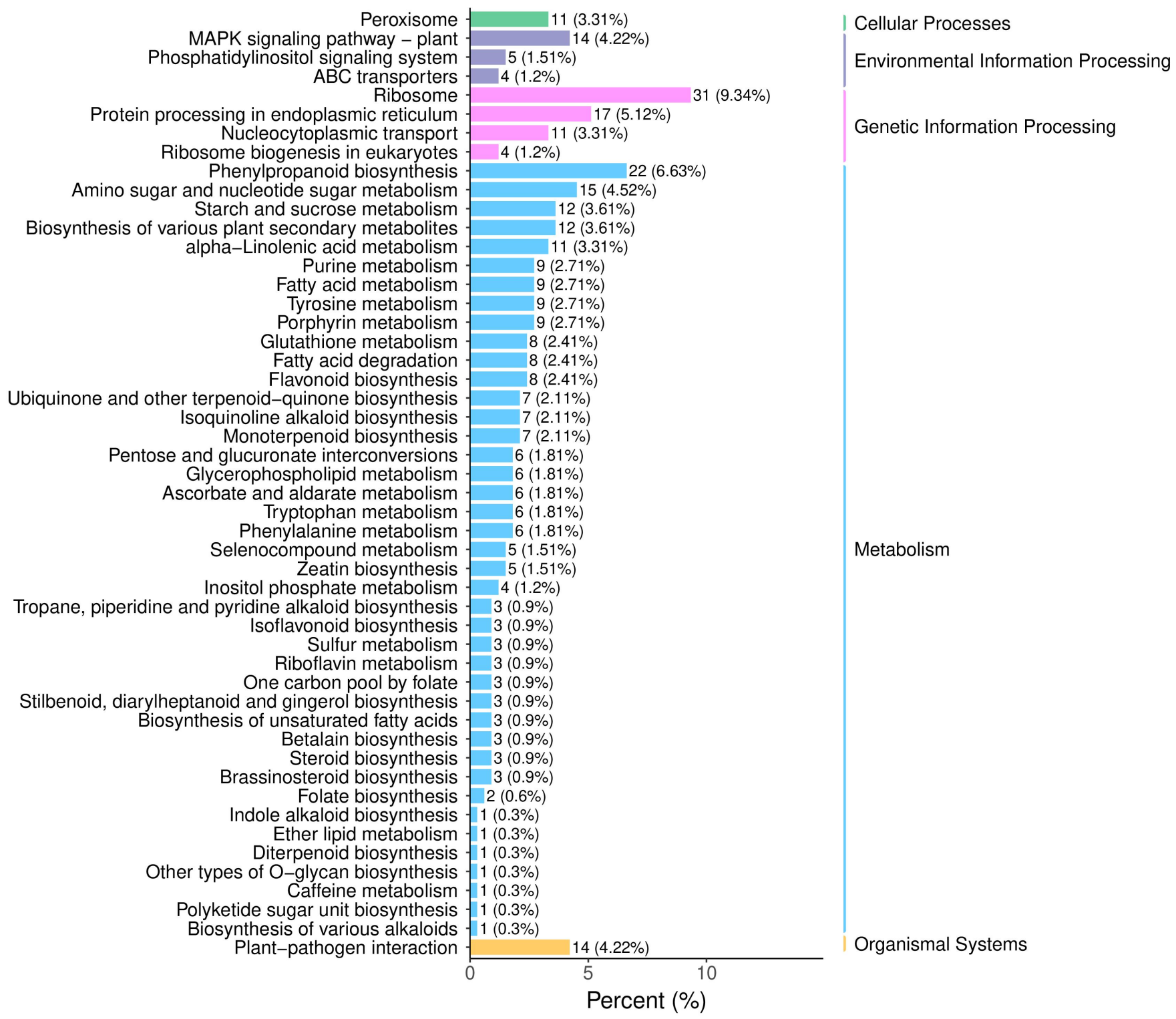
Peroxisome
11 (3.31%)
MAPK signaling pathway − plant
14 (4.22%)
Phosphatidylinositol signaling system
5 (1.51%)
ABC transporters
4 (1.2%)
Ribosome
31 (9.34%)
Protein processing in endoplasmic reticulum
17 (5.12%)
Nucleocytoplasmic transport
11 (3.31%)
Ribosome biogenesis in eukaryotes
4 (1.2%)
Phenylpropanoid biosynthesis
22 (6.63%)
Amino sugar and nucleotide sugar metabolism
15 (4.52%)
Starch and sucrose metabolism
12 (3.61%)
Biosynthesis of various plant secondary metabolites
12 (3.61%)
alpha−Linolenic acid metabolism
11 (3.31%)
Purine metabolism
9 (2.71%)
Fatty acid metabolism
9 (2.71%)
Tyrosine metabolism
9 (2.71%)
Porphyrin metabolism
9 (2.71%)
Glutathione metabolism
8 (2.41%)
Fatty acid degradation
8 (2.41%)
Flavonoid biosynthesis
8 (2.41%)
Ubiquinone and other terpenoid−quinone biosynthesis
7 (2.11%)
Isoquinoline alkaloid biosynthesis
7 (2.11%)
Monoterpenoid biosynthesis
7 (2.11%)
Pentose and glucuronate interconversions
6 (1.81%)
Glycerophospholipid metabolism
6 (1.81%)
Ascorbate and aldarate metabolism
6 (1.81%)
Tryptophan metabolism
6 (1.81%)
Phenylalanine metabolism
6 (1.81%)
Selenocompound metabolism
5 (1.51%)
Zeatin biosynthesis
5 (1.51%)
Inositol phosphate metabolism
4 (1.2%)
Tropane, piperidine and pyridine alkaloid biosynthesis
3 (0.9%)
Isoflavonoid biosynthesis
3 (0.9%)
Sulfur metabolism
3 (0.9%)
Riboflavin metabolism
3 (0.9%)
One carbon pool by folate
3 (0.9%)
Stilbenoid, diarylheptanoid and gingerol biosynthesis
3 (0.9%)
Biosynthesis of unsaturated fatty acids
3 (0.9%)
Betalain biosynthesis
3 (0.9%)
Steroid biosynthesis
3 (0.9%)
Brassinosteroid biosynthesis
3 (0.9%)
Folate biosynthesis
2 (0.6%)
Indole alkaloid biosynthesis
1 (0.3%)
Ether lipid metabolism
1 (0.3%)
Diterpenoid biosynthesis
1 (0.3%)
Other types of O−glycan biosynthesis
1 (0.3%)
Caffeine metabolism
1 (0.3%)
Polyketide sugar unit biosynthesis
1 (0.3%)
Biosynthesis of various alkaloids
1 (0.3%)
Plant−pathogen interaction
14 (4.22%)
0
5
10
Percent (%)
Cellular Processes
Environmental Information Processing
Genetic Information Processing
Metabolism
Organismal Systems

(C)

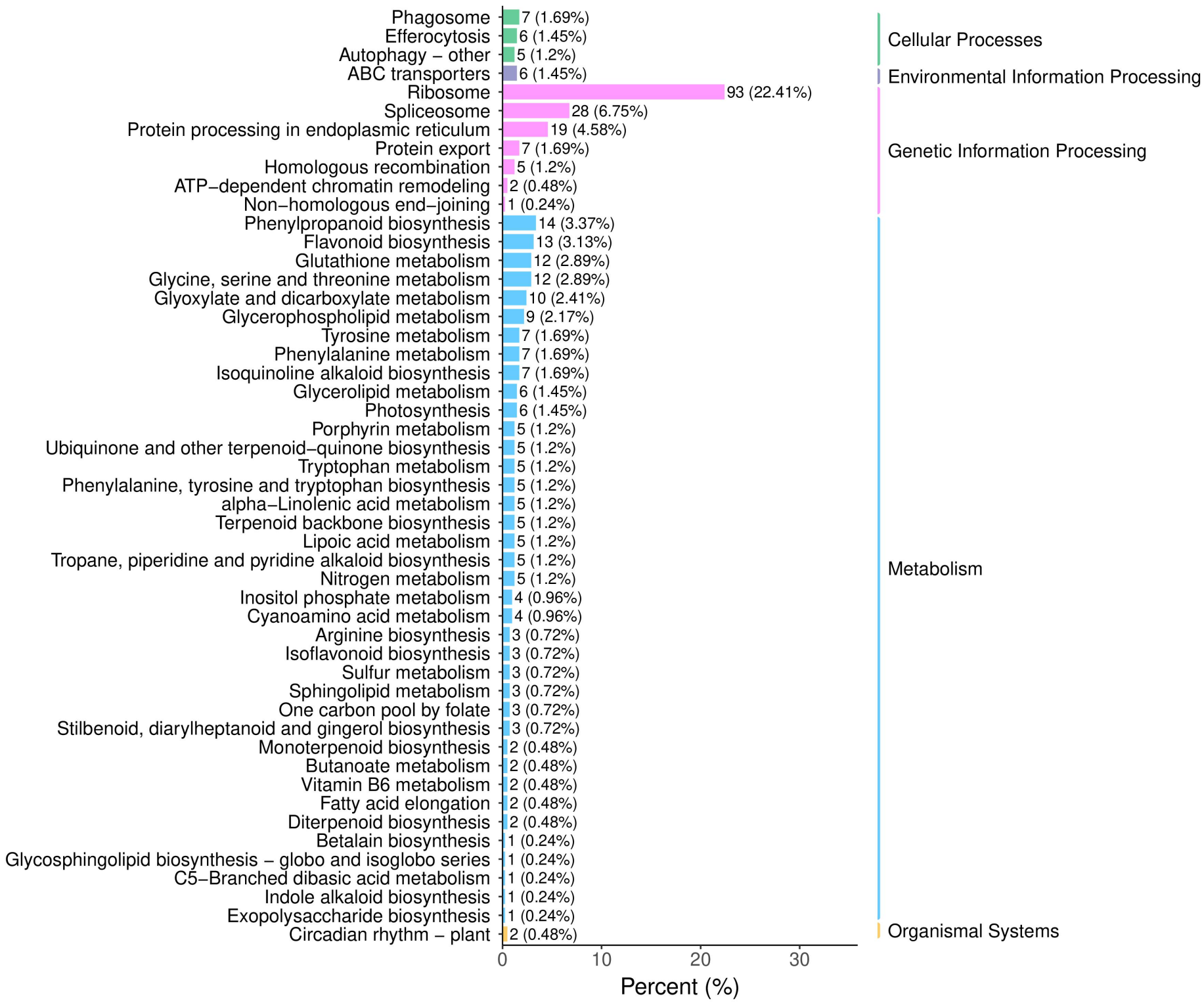

(D)

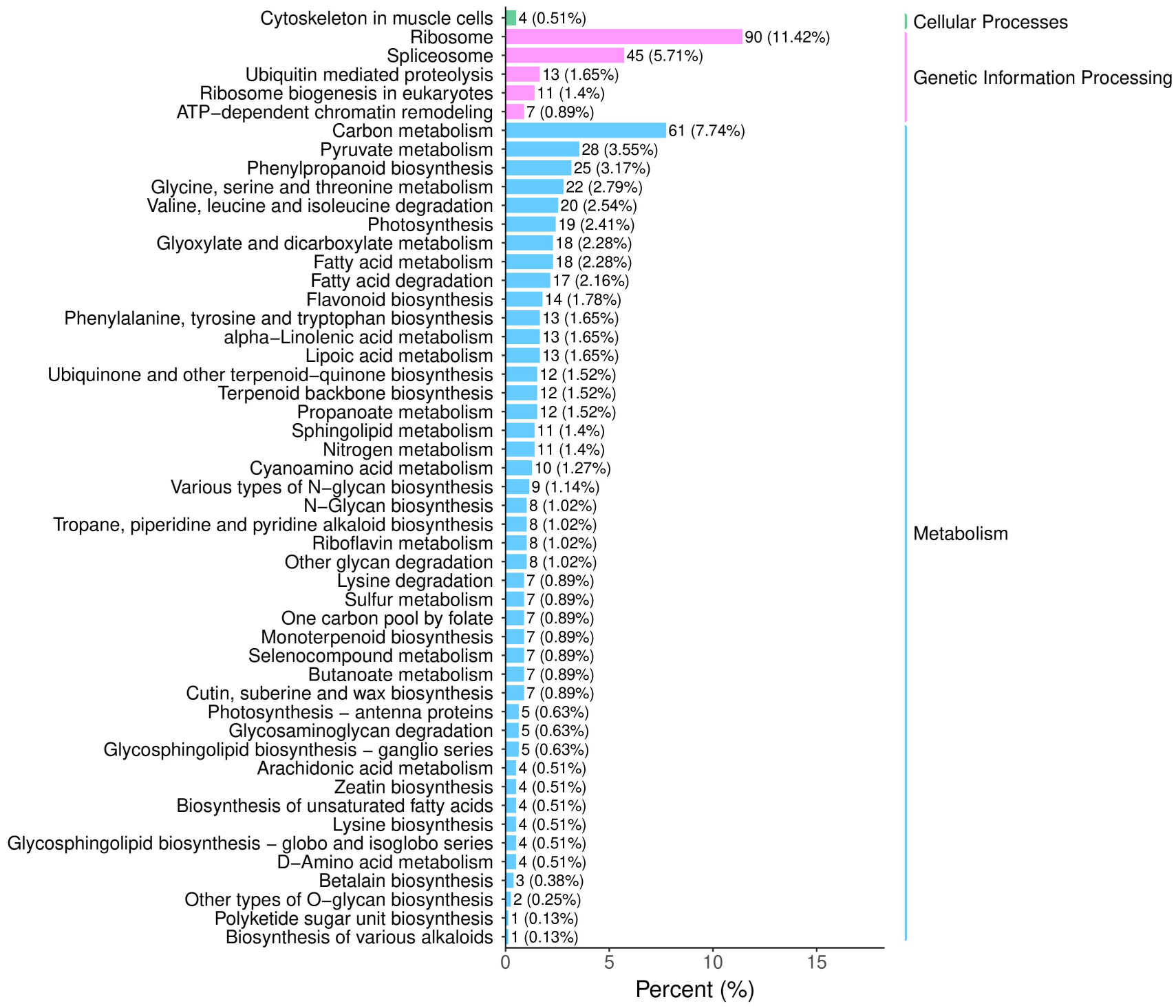

Supplementary Figure 4 KEGG pathway enrichment analysis of DEPs across four pairwise comparisons in *R. yedoense* var. *poukhanense*. Pathways are grouped into Cellular Processes, Environmental Information Processing, Genetic Information Processing, Metabolism, and Organismal Systems. Bar length indicates the percentage

of enriched DEPs within each pathway; numbers in parentheses show the absolute DEP count. (A) LT vs CK; (B) A vs CK; (C) ALT vs LT; (D) ALT vs A.
