# Supplementary material for: Exogenous ABA enhances cold tolerance of Rhododendron yedoense var. poukhanense under subzero temperature: integrating physiology, transcriptome, and proteome
Source: Front Plant Sci. 2026 Aug 28;17:1921248. doi: 10.3389/fpls.2026.1921248 (PMC13561794; doi:10.3389/fpls.2026.1921248)
Supplement: Supplementary file 5 [file DataSheet5.pdf]

(A)

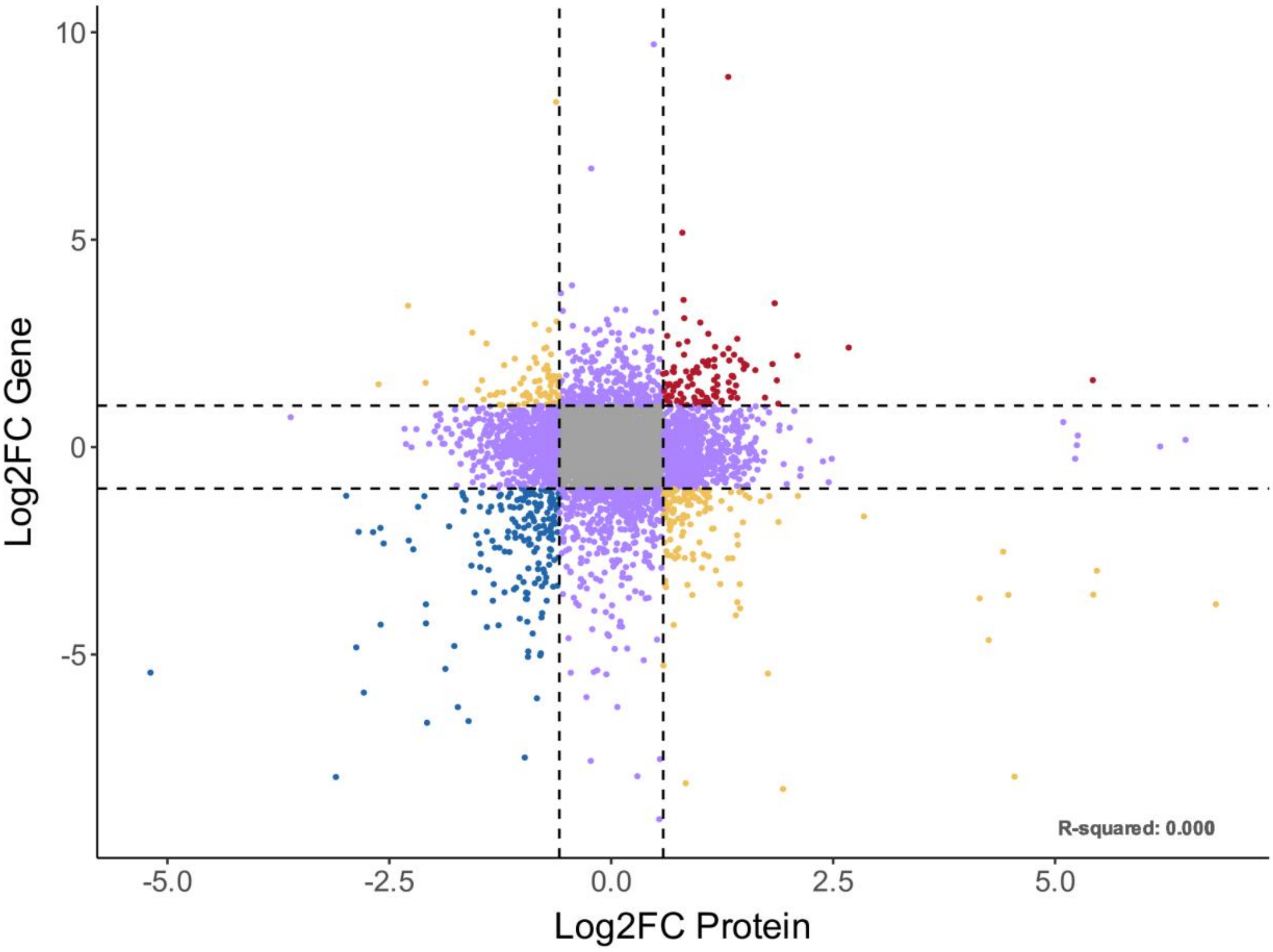

Log2FC Gene
10
5
0
-5
R-squared: 0.000
-5.0
-2.5
0.0
2.5
5.0
Log2FC Protein

(B)

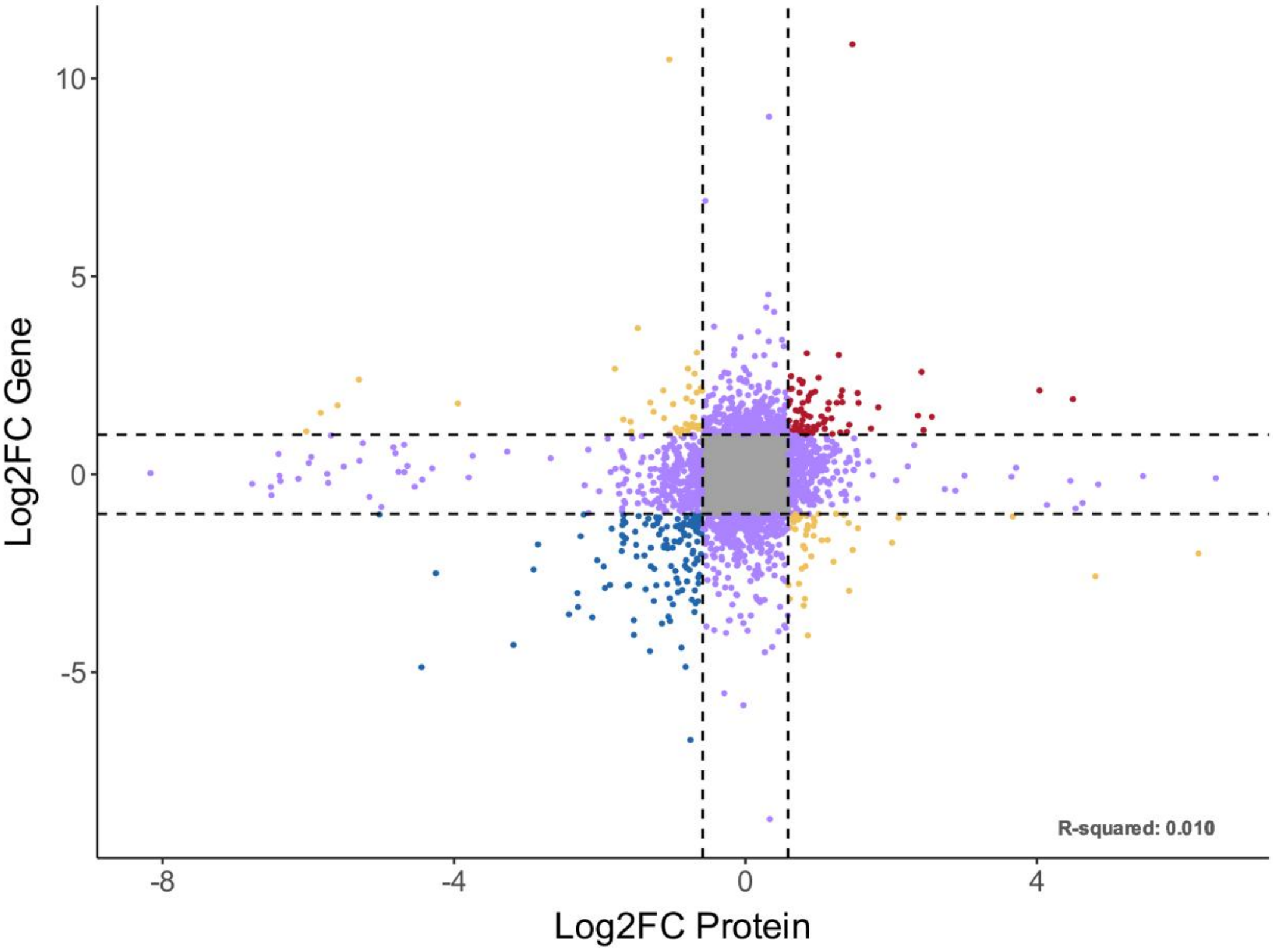

Log2FC Gene
10
5
0
-5
R-squared: 0.010
-8
-4
0
4
Log2FC Protein

(C)

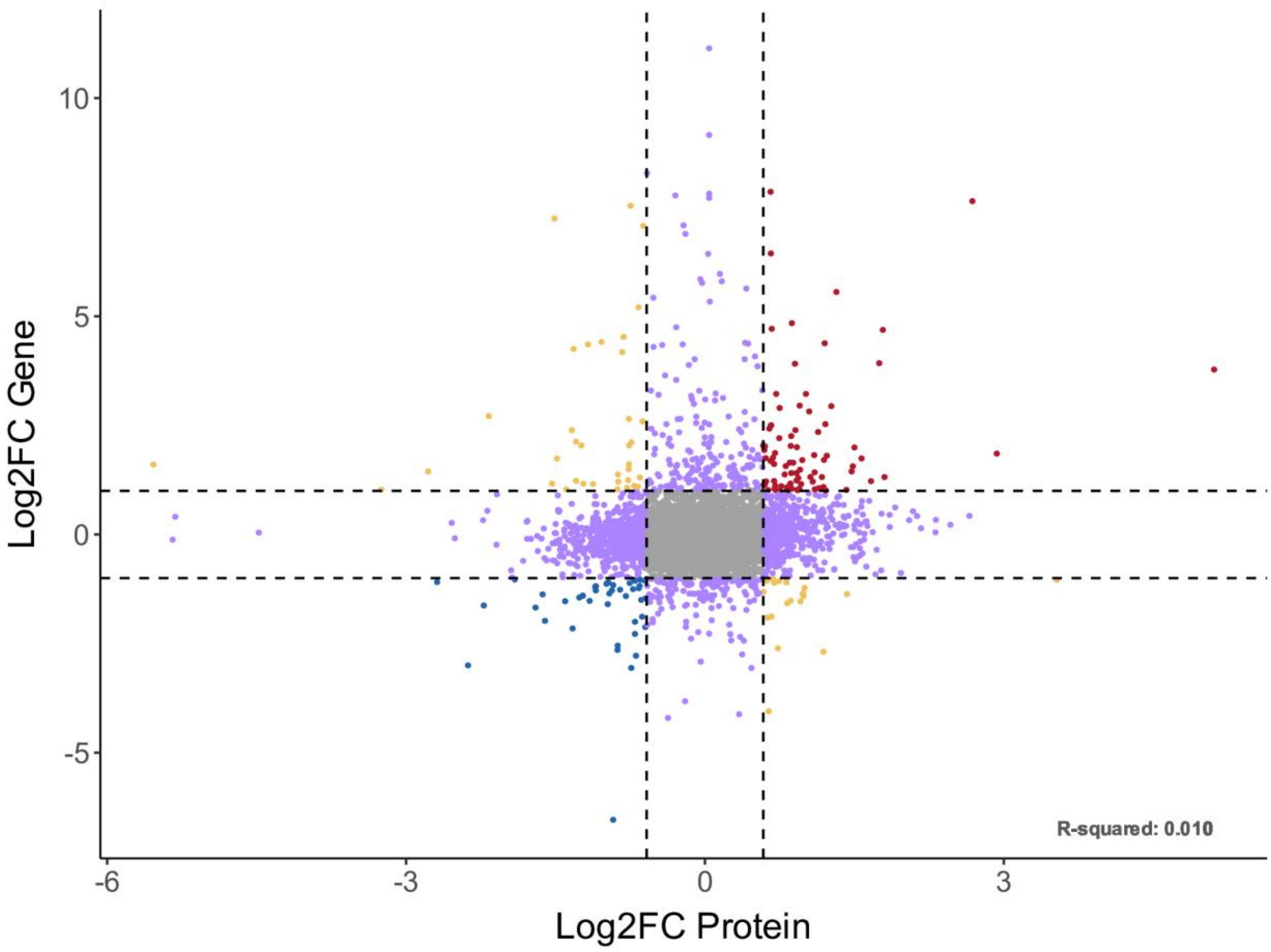

(D)

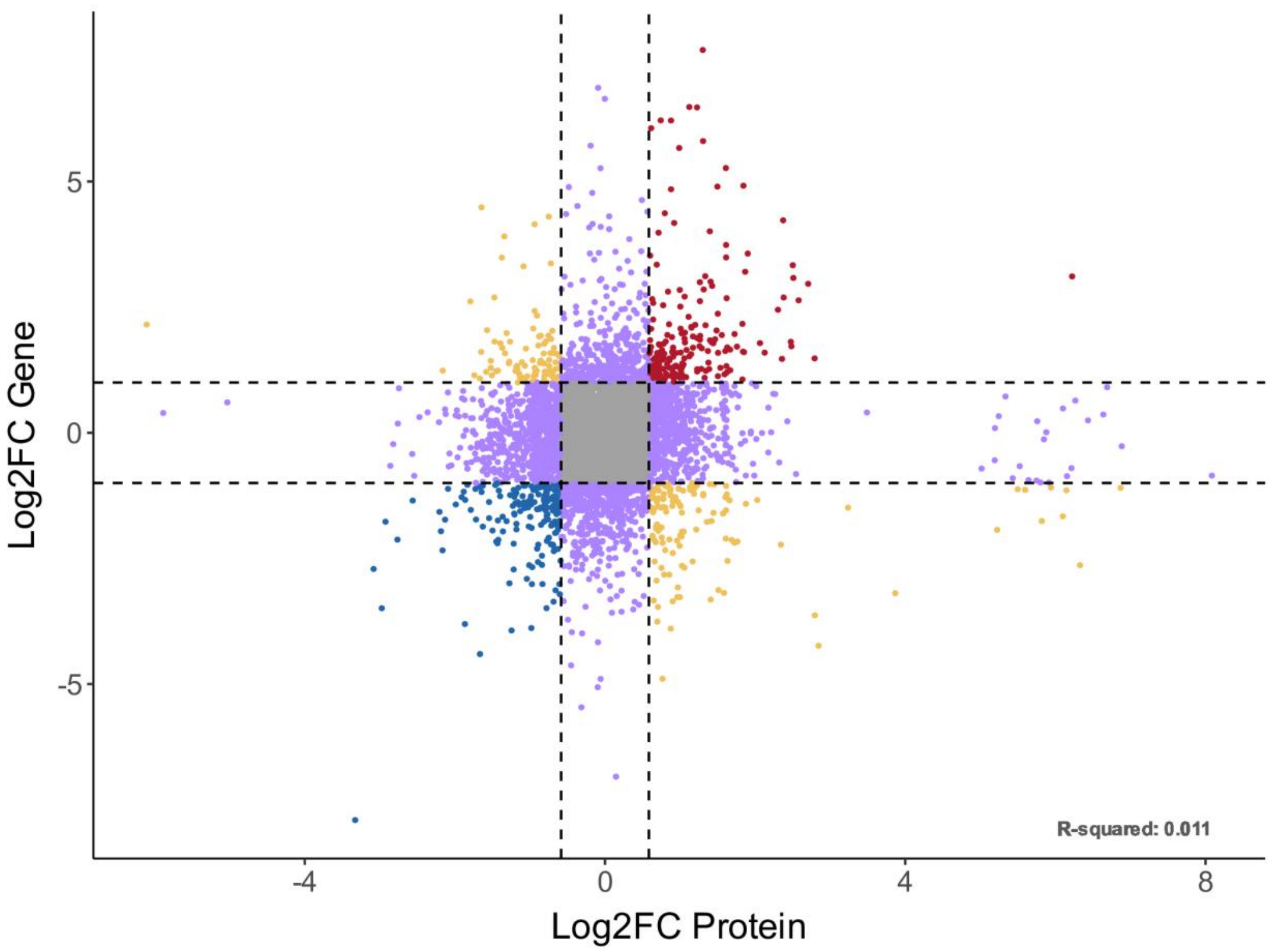

Supplementary Figure 5 Nine-quadrant analysis of transcriptome-proteome correlation across four pairwise comparisons in *R. yedoense* var. *poukhanense*. Scatter

plots show $\log_2$ fold change of proteins (x-axis) versus corresponding genes (y-axis). Dashed lines indicate significance thresholds ($|\log_2FC| \geq 1$ for transcriptome, $|\log_2FC| \geq 0.263$ for proteome). Quadrant colors: red = both up-regulated (Q1); dark blue = both down-regulated (Q9); purple = transcript changed only; yellow = protein changed only; grey = neither changed (Q5). Pearson correlation coefficients (r) and concordance rates are indicated. (A) LT vs CK, r = 0.016, concordance 4.1%; (B) A vs CK, r = 0.101, concordance 1.7%; (C) ALT vs LT, r = 0.101, concordance 1.0%; (D) ALT vs A, r = 0.104, concordance 4.1%. The consistently weak correlations across all comparisons demonstrate pervasive post-transcriptional regulation decoupling transcript abundance from protein levels.
