# Supplementary material for: Exogenous ABA enhances cold tolerance of Rhododendron yedoense var. poukhanense under subzero temperature: integrating physiology, transcriptome, and proteome
Source: Front Plant Sci. 2026 Aug 28;17:1921248. doi: 10.3389/fpls.2026.1921248 (PMC13561794; doi:10.3389/fpls.2026.1921248)
Supplement: Supplementary file 6 [file DataSheet6.pdf]

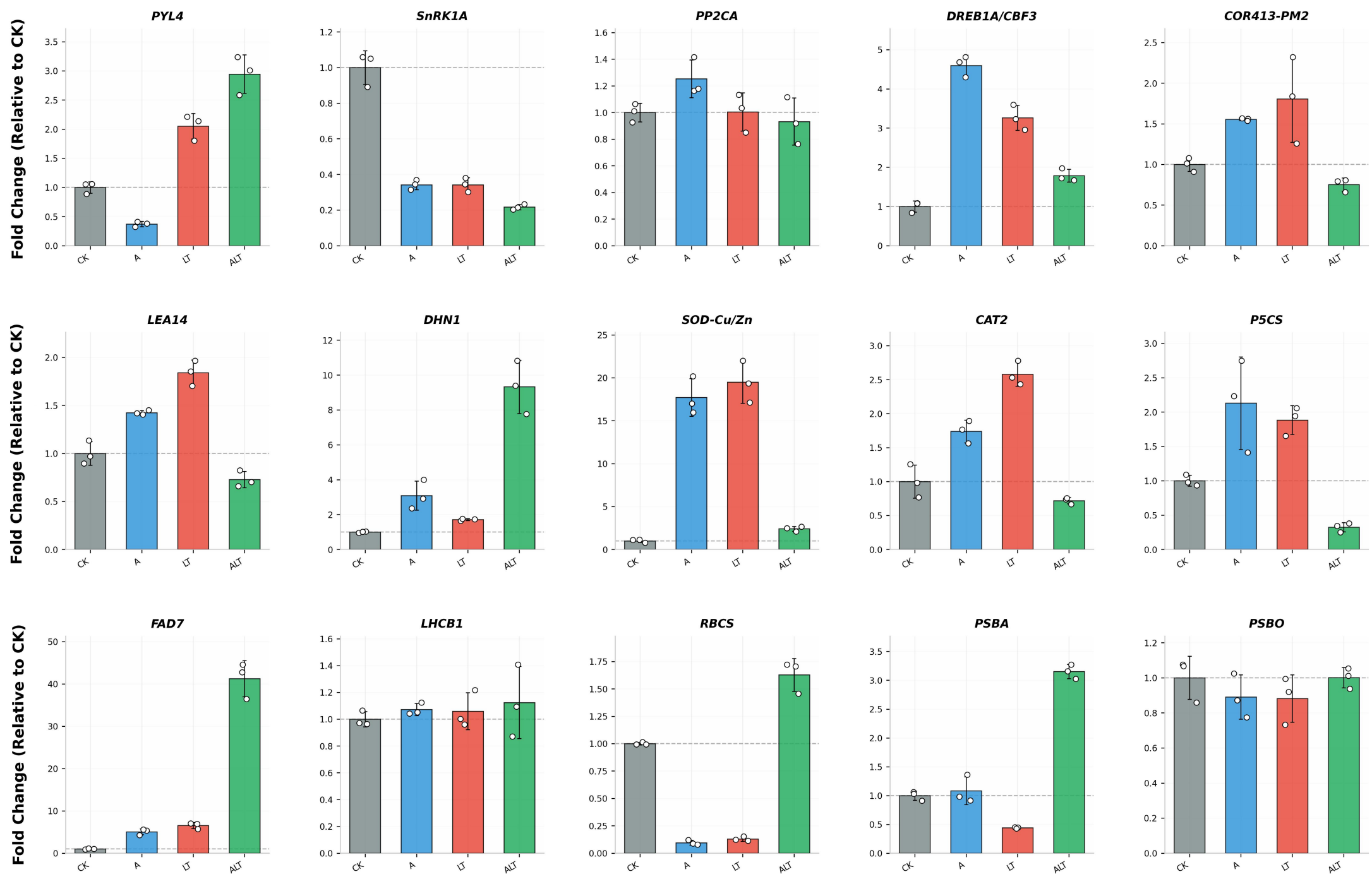

Supplementary Figure 6 qRT-PCR validation of RNA-seq results for 15 DEGs across CK, A, LT and ALT of *R. yedoense* var. *poukhanense*. qRT-PCR-derived fold change values ($2^{\wedge-\Delta\Delta Ct}$) for 15 differentially expressed genes, with CK set as the calibrator (fold change = 1.0, dashed line). Values above 1.0 indicate higher expression than CK; values below 1.0 indicate lower expression. Bars represent mean ± SEM (n = 3 biological replicates). Open circles show individual data points. Gene names are italicized per botanical convention. Grey bars, CK; blue bars, A; Red bars, LT; green bars, ALT.
