# Supplementary material for: Exogenous ABA enhances cold tolerance of Rhododendron yedoense var. poukhanense under subzero temperature: integrating physiology, transcriptome, and proteome
Source: Front Plant Sci. 2026 Aug 28;17:1921248. doi: 10.3389/fpls.2026.1921248 (PMC13561794; doi:10.3389/fpls.2026.1921248)
Supplement: Supplementary file 7 [file DataSheet7.pdf]

Supplementary Table 1 qRT-PCR primer sequences used for validation of RNA-seq data.

No.	Gene Symbol	Forward Primer (5'→3')	Reverse Primer (5'→3')
1	*PYL4*	TGGTTCAAATGGGCACGAAAG	TTGTGCAGTTGCTTGTAGGC
2	*SnRK1A*	GGAATCACGGAAGAAAGCC	AAGGAGGGTTTCCAAGGAAG
3	*PP2CA*	CATGATAGTGAGAGAGGAGG	GTTTCGAGATCCAACTGCAG
4	*DREB1A/CBF3*	TGGGAGTTGGAGGAACATTG	AGCTTAAAGTCAGGAATGCTC
5	*COR413-PM2*	CCGATGCTGAAATGATCGAC	CCTCCTTTGCTGCTATTTAC
6	*LEA14*	TATCGGGGAGTTGCACTAGC	ACACTCACCTCATAGTGTCC
7	*DHN1*	TGGCACAATATGGGAGTGG	TTCACCACACGGGTACTATG
8	*SOD-Cu/Zn*	TCGCTACTGAAACTCCCAAG	ACGAAGCGGAGAAATCACTG
9	*CAT2*	AGCACCACCATATAAGCCAC	ACAAGCTCCGGTTAATCCAG
10	*P5CS*	ATCATCTTCGCCTCCATCAC	GAGGCTTCGGCTGTTTCC
11	*FAD7*	GTTGGGGAATCGAAGCTTTC	GAGAAGAAAGGAGAAGGGAAG
12	*LHCB1*	CTACTCCATCCTTTCACTCG	GAGGAGAGCAACGATTATGC
13	*RBCS*	GTGCGGCAAAAACTTGGTAC	ACGTGCTTTTGGGGAAAACG
14	*PSBA*	CATGATCGTATTCCAGGCTG	TTTGATCAGGGAAACCACCG
15	*PSBO*	CCATGAACGCAGTCTACAAC	TCGGGTGGCAACAGTACTTC
16	*EF1-α*	ATTGGCCATGTTGACTCTGG	ACCCAGGCGTACTTGAATGA
