# Supplementary material for: Exogenous ABA enhances cold tolerance of Rhododendron yedoense var. poukhanense under subzero temperature: integrating physiology, transcriptome, and proteome
Source: Front Plant Sci. 2026 Aug 28;17:1921248. doi: 10.3389/fpls.2026.1921248 (PMC13561794; doi:10.3389/fpls.2026.1921248)
Supplement: Supplementary file 8 [file DataSheet8.pdf]

**Supplementary Table 2** qRT-PCR validation of 15 selected DEGs.

| No. | Gene ID | Gene Symbol | Full Name | Functional Category | RNA-seq $log_2FC$ (LT vs CK) | RNA-seq $log_2FC$ (A vs CK) | RNA-seq $log_2FC$ (ALT vs CK) | RNA-seq $log_2FC$ (ALT vs LT) | qPCR $log_2FC$ (LT vs CK) | qPCR $log_2FC$ (A vs CK) | qPCR $log_2FC$ (ALT vs CK) | qPCR $log_2FC$ (ALT vs LT) |
|---|---|---|---|---|---|---|---|---|---|---|---|---|
| 1 | RHSIM_Rhsim01G0255800 | *PYL4* | PYR/PYL/RCAR-type receptor 4 | ABA signal reception | 2.135 | 2.18 | -0.83 | -1.978 | 1.036 | 1.558 | -1.099 | -1.077 |
| 2 | RHSIM_Rhsim12G0097400 | *SnRK1A* | SNF1-related protein kinase 1A | ABA signal transduction | 0.781 | 1.441 | 1.809 | 1.942 | -1.426 | -1.547 | -2.207 | -2.246 |
| 3 | RHSIM_Rhsim03G0133900 | *PP2CA* | Protein phosphatase 2C A | ABA signal attenuation | -1.589 | -1.58 | 0.726 | 0.842 | N/A | N/A | N/A | N/A |
| 4 | RHSIM_Rhsim10G0156500 | *DREB1A/CBF3* | Dehydration-responsive element-binding protein 1A | Transcription regulation | 0.868 | 1.383 | -0.603 | -1.119 | 1.706 | 2.201 | 0.838 | 1.132 |
| 5 | RHSIM_Rhsim08G0109600 | *COR413-PM2* | Cold-regulated 413 plasma membrane protein 2 | Cold acclimation/LEA | 1.263 | 1.758 | -0.357 | -1.972 | 0.852 | 0.637 | -0.411 | -0.866 |
| 6 | RHSIM_Rhsim06G0028600 | *LEA14* | Late embryogenesis abundant protein 14 | Dehydration protection | 1.339 | 1.796 | 0.554 | 0.32 | 0.88 | 0.51 | -0.459 | -1.089 |
| 7 | RHSIM_Rhsim08G0122300 | *DHN1* | Dehydrin DHN1 | Dehydration response | -2.447 | -4.004 | 2.118 | 0.871 | 0.775 | 1.626 | 3.221 | 2.015 |
| 8 | RHSIM_Rhsim07G0068300 | *SOD-Cu/Zn* | Superoxide dismutase [Cu-Zn] | Antioxidant defense | 3.02 | 2.879 | N/A | N/A | 4.284 | 4.147 | 1.264 | 1.603 |
| 9 | RHSIM_Rhsim11G0104000 | *CAT2* | Catalase isozyme 2 | $H_2O_2$ detoxification | 1.843 | 2.717 | 0.612 | -0.227 | 1.368 | 0.798 | -0.476 | -0.155 |
| 10 | RHSIM_Rhsim10G0024000 | *P5CS* | Delta-1-pyrroline-5-carboxylate synthetase | Proline biosynthesis | 2.546 | 3.018 | N/A | N/A | 0.914 | 1.091 | -1.633 | -1.46 |
| 11 | RHSIM_Rhsim12G0023500 | *FAD7* | Omega-3 fatty acid desaturase | Membrane lipid unsaturation | -2.656 | -2.109 | -1.003 | -1.093 | 2.712 | 2.343 | 5.367 | 6.146 |
| 12 | RHSIM_Rhsim02G0223700 | *LHCB1* | Light-harvesting complex B1 | Photosynthesis (down) | -3.167 | -4.05 | -5.193 | -4.34 | N/A | N/A | N/A | N/A |
| 13 | RHSIM_Rhsim07G0131400 | *RBCS* | Ribulose bisphosphate carboxylase small chain | Carbon fixation (down) | -3.644 | -2.799 | 0.324 | 1.482 | -2.941 | -3.385 | 0.703 | 0.481 |
| 14 | RHSIM_RhsimPtG0005800 | *PSBA* | Photosystem II protein D1 | Photosystem II core (down) | -2.84 | -3.09 | -0.992 | -1.222 | -1.184 | 0.117 | 1.656 | 2.055 |
| 15 | RHSIM_Rhsim09G0165900 | *PSBO* | Photosystem II oxygen-evolving enhancer protein 1 | Water splitting (down) | -3.878 | -2.126 | 1.324 | 1.626 | N/A | N/A | N/A | N/A |

Note: RNA-seq $log_2FC = log_2$ fold change from RNA sequencing; qPCR $log_2FC = log_2$fold change from quantitative real-time PCR calculated as $log_2(2^{-\Delta\Delta Ct})$. Positive values indicate upregulation; negative values indicate downregulation. N/A indicates no significant difference in that comparison.
